# Long-term ambient air pollution and the risk of major mental disorder: A prospective cohort study

**DOI:** 10.1192/j.eurpsy.2024.1809

**Published:** 2024-12-18

**Authors:** Chuyu Pan, Bolun Cheng, Shiqiang Cheng, Li Liu, Xuena Yang, Peilin Meng, Xin Qi, Na Zhang, Xiaoyue Qin, Dan He, Wenming Wei, Jingni Hui, Yan Wen, Yumeng Jia, Huan Liu, Feng Zhang

**Affiliations:** 1 Key Laboratory of Trace Elements and Endemic Diseases of National Health and Family Planning Commission, Key Laboratory of Environment and Genes Related to Diseases of Ministry of Education of China, Key Laboratory for Disease Prevention and Control and Health Promotion of Shaanxi Province, School of Public Health, Health Science Center, Xi’an Jiaotong University, Xi’an, China; 2 Precision Medicine Center, The First Affiliated Hospital of Xi’an Jiaotong University, Xi’an, China

**Keywords:** air pollution, genetic susceptibility, income, major mental disorder, social deprivation

## Abstract

**Background:**

Despite growing awareness of the mental health damage caused by air pollution, the epidemiologic evidence on impact of air pollutants on major mental disorders (MDs) remains limited. We aim to explore the impact of various air pollutants on the risk of major MD.

**Methods:**

This prospective study analyzed data from 170 369 participants without depression, anxiety, bipolar disorder, and schizophrenia at baseline. The concentrations of particulate matter with aerodynamic diameter ≤ 2.5 μm (PM_2.5_), particulate matter with aerodynamic diameter > 2.5 μm, and ≤ 10 μm (PM_2.5–10_), nitrogen dioxide (NO_2_), and nitric oxide (NO) were estimated using land-use regression models. The association between air pollutants and incident MD was investigated by Cox proportional hazard model.

**Results:**

During a median follow-up of 10.6 years, 9 004 participants developed MD. Exposure to air pollution in the highest quartile significantly increased the risk of MD compared with the lowest quartile: PM_2.5_ (hazard ratio [HR]: 1.16, 95% CI: 1.09–1.23), NO_2_ (HR: 1.12, 95% CI: 1.05–1.19), and NO (HR: 1.10, 95% CI: 1.03–1.17). Subgroup analysis showed that participants with lower income were more likely to experience MD when exposed to air pollution. We also observed joint effects of socioeconomic status or genetic risk with air pollution on the MD risk. For instance, the HR of individuals with the highest genetic risk and highest quartiles of PM_2.5_ was 1.63 (95% CI: 1.46–1.81) compared to those with the lowest genetic risk and lowest quartiles of PM_2.5_.

**Conclusions:**

Our findings highlight the importance of air pollution control in alleviating the burden of MD.

## Introduction

Mental disorders (MDs) are experiencing a widespread global prevalence [1]. According to Global Burden of Disease (GBD) Study 2019, there are about 970 million people with MD worldwide, and MDs have become the seventh leading cause of disability-adjusted life-years (DALYs) [1]. Dealing with mental illness and its impact on individual health and the global economy is confronted with formidable challenges [1, 2]. Addressing these issues requires the identification of manageable risk factors for MD, which is vital for their prevention and effective management.

Air pollution has emerged as a major risk factor contributing to the global burden of disease [3]. In recent years, the neurotoxicity of air pollution has garnered increased attention, such as inducing oxidative stress and inflammation in the central nervous system (CNS), which leads to neuronal damage [4]. Previous studies have identified associations between air pollution and an increased risk of single MD, such as depression [5] and anxiety disorders [6]. MDs often exhibit co-occurrences and share pathophysiological commonalities [7]. For instance, reduced neocortical thickness and similar neurotransmitter receptor profiles have been observed across various MDs [8, 9]. Additionally, shared environmental factors such as childhood abuse and residential mobility during upbringing further underscore the commonality of these disorders [7, 9]. Considering the neurotoxic effects of air pollution on the CNS (e.g., oxidative stress and inflammation) [4], air pollution may not act as a specific risk factor for a single mental illness. Previous researches predominantly focused on the impact of air pollutants on single, or at most two, MDs such as depression and anxiety [5, 6]. However, to date, there is a dearth of large-scale prospective studies investigating whether long-term air pollution serves as a general psychopathological factor and its overall impact on MD incidence. Understanding the extent and public health significance of cross-disorder influences of air pollution is crucial for formulating comprehensive risk prediction, prevention, and control strategies for MDs.

The level of exposure to air pollution is associated with socioeconomic inequality [10]. Socioeconomic status, such as household income and deprivation, was also associated with mental health [11, 12]. Preliminary evidence suggested that the associations between air pollution and the risk of depression varied among individuals with different socioeconomic backgrounds. In the French CONSTANCES cohort study, a stronger adverse association between air pollution and depressive symptoms was observed in males with low income and higher community deprivation, and significant interactions were detected between socioeconomic status and air pollution [13]. However, the influence of socioeconomic status on the associations between air pollution and major MD remains to be fully understood.

MDs are moderately to highly heritable [14]. The combined effect of air pollution and genetic risk has been found to significantly increase the risk of individual MD [6, 15]. However, the effects of interactions between polygenic risk scores (PRSs) and air pollutions on MD risk remain controversial [6, 15]. The contribution of genetic loci to MD is pleiotropic [16], estimating the overall genetic risk of multiple genetically associated MD is more helpful in identifying individuals with shared predispositions for MD [17]. To date, the role of genetic susceptibility in the effects of air pollution on overall mental illness remains unknown. In order to investigate this effect, it is essential to estimate multitrait genetic risk [17] estimates for MD.

Based on the large-scale UK Biobank cohort data, we explored the impact of various air pollutants on the risk of overall major MD, including depression, anxiety disorder, schizophrenia, and bipolar disorder, which represent some of the most prevalent and impactful MDs [1]. We further investigated the influence of socioeconomic status on the association between air pollution and major MD. Moreover, we constructed the multitrait PRS (mt-PRS), which can predict multitrait genetic risk for overall MD by integrating information on multiple genetically related traits [17] and examined the impact of air pollution on major MD across different genetic risk levels.

## Methods

### Study population and design

This prospective study was conducted based on UK Biobank. UK Biobank is a large-scale population-based prospective study, which contains biological samples and phenotype data from more than 500 000 people aged 40–69 years assessed between 2006 and 2010, encompassing 22 assessment centers across the United Kingdom. During this period, participants completed self-administered touch-screen questionnaires and underwent brief computer-assisted interviews as part of their assessment visit. Ethical approval for the UK Biobank study was obtained from the North West Multi-Center Research Ethics Committee, under approval number 11/NW/0382. All participants provided informed consent, granting UK Biobank access to their health-related records [18].

**Figure 1.**
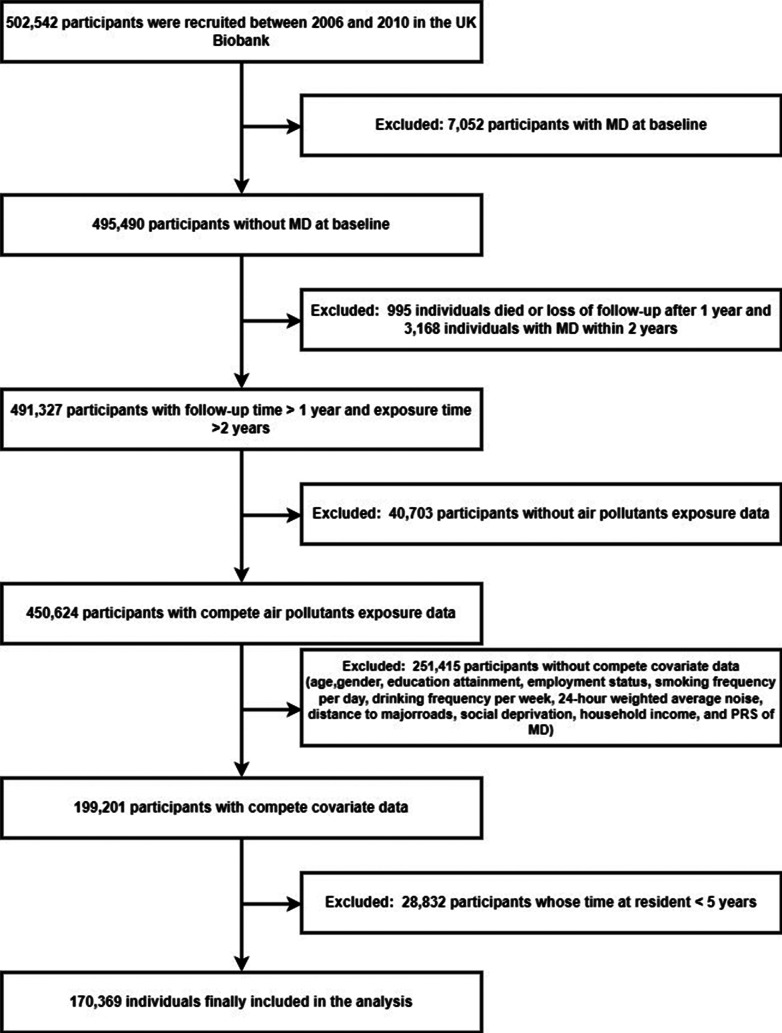
Flow chart of participants included in the study.

In this study, we excluded individuals with diagnoses of depression, anxiety, schizophrenia, and bipolar disorder at baseline. The diagnostic criteria used for this exclusion were based on the *International Classification of Diseases*, 10th Revision (ICD-10). In addition, we excluded individuals without air pollution exposure data and covariate data, individuals who died within 1 year or lost to follow up, as well as individuals whose time at residence less than 5 years. To avoid reverse causation, individuals diagnosed with MD within 2 years of the start of follow-up were also excluded (Figure 1).

### Outcomes and follow-up

During the follow-up period, occurrences of depression, anxiety disorders, schizophrenia, and bipolar disorder were defined as MD according to the ICD-10. Depression was defined based on ICD-10 codes F32–F33, anxiety disorders on F40–F41, schizophrenia on F20–F29, and bipolar disorder on F31. The follow-up time was calculated from the baseline date to the diagnosis of the disease outcome, death, loss to follow up, and the end of the follow-up on December 31, 2019, whichever occurred first. For individuals diagnosed with multiple MDs, the earlier time was considered the diagnosis time. Participants who received a diagnosis of MDs before the deadline were coded as 1, while those who did not experience the disease or death and loss to follow up before the deadline were coded as 0. Detailed definitions of MDs are provided in the Supplementary Materials.

### Air pollutants

The air pollution data were derived from the European Study of Cohorts for Air Pollution Effects (ESCAPE) project, which covered 20 European regions for particulate matter (PM) research and 32 European regions for nitrogen dioxide (NO_2_) and nitrogen oxides (NO_x_) research [19, 20]. Land-use regression (LUR) models were used to assess individual participants’ residential air pollution exposure based on their home addresses [19, 20]. In this study, NO_2_, nitric oxide (NO), PM_2.5_ (particulate matter with aerodynamic diameter ≤ 2.5 μm), and PM_2.5–10_ (2.5 μm < particulate matter with aerodynamic diameter ≤ 10 μm) were used as the primary air pollution indicators. The exposure levels for NO_2,_ PM_2.5_, and PM_2.5–10_ were determined using the 2010 annual average concentrations. The exposure levels for NO were estimated by subtracting the NO_2_ concentration in 2010 from the NO_x_ concentration in 2010 [21].

### Socioeconomic status

The socioeconomic status was evaluated according to household income and the social deprivation level, respectively. Participants with annual household income below £18 000 were classified as having low income, those with income between £18 000 and £51 999 were categorized as having moderate income, and individuals with income exceeding £51 999 were considered to have high income. Social deprivation level was defined based on the Townsend Deprivation Index (TDI) and stratified into tertiles. Individuals with TDI in the lowest tertile were categorized as low deprivation, those in the middle tertile as moderate deprivation, and those in the highest tertile as high deprivation.

### Mt-PRS

The mt-PRS was employed based on principal component analysis (PCA) weighting to assess the polygenic risk of MD [17]. The mt-PRS combines PRS from multiple traits with weights obtained from the genetic correlation matrix derived through PCA [17]. First, we utilized linkage disequilibrium score regression (LDSC) to compute the genetic correlations for depression, anxiety disorders, schizophrenia, and bipolar disorder. Subsequently, PCA was applied to the genetic correlation matrix, and the weights were computed based on eigenvectors with eigenvalues greater than 1, and explaining at least 90% of the variance [22, 23]. PRSice-2 [24] was then employed to calculate PRS for depression, anxiety disorders, schizophrenia, and bipolar disorder, using publicly available Genome-wide association study (GWAS) summary data and genotypes of UK Biobank participants. Finally, the mt-PRS was calculated using the sum of the weighted individual PRSs. Detailed method was provided in the Supplementary Materials.

### Covariates

In this study, basic demographic variables (gender, age), socioeconomic indicators (education level, employment status, social deprivation, household income), genetic risk of MD, lifestyle factors (smoking and drinking frequency), and local environmental exposures (24-h weighted average noise, distance to major roads) were considered as covariates. The age was categorized into <65 years and ≥ 65 years. The smoking frequency was defined according to the maximum number of reported past or current cigarettes consumed per day, and the drinking frequency was measured by the sum of all alcoholic drinks per week [25]. Education attainment was synthesized into “university or college degree” and “others.” Employment status was categorized into active (in paid) or inactive (not employed or not in paid). The genetic risk of MD was defined based on the tertiles of mt-PRS. The 24-h weighted average noise was calculated with a 5- and 10-decibel (dB) penalty added to evening and night time, respectively. Proximity to major road was indicated by the inverse distance to the nearest major road. Additionally, diet and exercise were considered in the sensitivity analysis. The diet was assessed using a healthy diet score based on the Mediterranean diet and heart-healthy dietary recommendations for reducing the risk of chronic diseases [26, 27], which included seven components: fruits, vegetables, fish, processed meat, unprocessed red meat, whole grains, and refined grains. Exercise was evaluated using International Physical Activity Questionnaire (IPAQ), and participants were classified into three levels of physical activity groups: high, moderate, and low [28]. The detailed definitions are shown in Supplementary Materials.

### Statistical analysis

Cox proportional hazards regression model was employed to explore the association between air pollution and MD using the “survival” package in R 4.1.0. Individual exposures to each air pollutant were stratified into quartiles based on their concentrations. Model 1 adjusted for gender and age. Model 2 additionally controlled for education attainment, employment status, smoking frequency per day, drinking frequency per week, 24-h weighted average noise, and distance to major roads. Model 3 was the main model, which further adjusted for social deprivation, household income, and genetic risk of MD, in addition to the covariates from Model 2. Except for the analysis of PM_2.5–10_, all other models satisfied the proportional hazard assumption. For PM_2.5–10_, age did not meet the proportional hazard assumption and was therefore adjusted as a stratification variable (<65 years and ≥65 years) in the analysis. We conducted sensitivity analysis by further adjusting healthy diet score and IPAQ activity. Moreover, additional analysis was performed to test the air pollution exposures with single MD.

To explore the impact of socioeconomic status and genetic risk on the association between air pollution and MD risk, we conducted subgroup analyses based on household income, social deprivation, and genetic risk of MD. Additionally, we assessed the joint effects of socioeconomic status or genetic risk with air pollution on MD risk by creating combination variables (air pollutants × income, air pollutants × social deprivation, and air pollutants × genetic risk of MD), resulting in 12 categories for each combination. A two-sided *P* < 0.05 was considered statistically significant.

## Results

### Demographic characteristics

Totally, 170 369 participants (mean [*SD*] age, 56.94 [7.75]) were included in the analysis, of whom 85 834 (50.4%) were males. During a median (interquartile range [IQR]) follow-up of 10.6 (10.0–11.3) years, 9 004 individuals were identified as MD cases. Among individuals with MD, 59.4% belonged to the low-income group at the baseline, 23.9% to the moderate-income group, and 16.7% to the high-income group. Additionally, 28.7% were in the low deprivation group, 31.5% in the moderate deprivation group, and 39.7% in the high deprivation group. Regarding genetic risk of MD, 28.0%, 32.9% and 39.1% had low, moderate, and high genetic risk, respectively. The median (IQR) concentration of pollutants are as follows: NO, 15.54 (11.24–20.02) μg/m^3^; NO_2_, 25.34 (20.77–30.23) μg/m^3^; PM_2.5_, 9.85 (9.21–10.46) μg/m^3^; and PM_2.5–10_, 6.08 (5.83–6.58) μg/m^3^. The demographic characteristic of participants is shown in Table 1.

**Table 1.** Demographic characteristic of participants

	Total (*N* = 170 369)	Individuals without mental disorder (*N* = 161 365)	Individuals with mental disorder (*N* = 9 004)
Gender			
Female	84 535(49.6)	78 951(48.9)	5 584(62.0)
Male	85 834 (50.4)	82 414 (51.1)	3 420 (38.0)
Age, year (mean [*SD*])	56.94 (7.75)	56.92 (7.75)	57.42 (7.80)
Employment			
Active	101 925 (59.8)	97 451 (60.4)	4 474 (49.7)
Inactive	68 444(40.2)	63 914(39.6)	4 530(50.3)
Education attainment			
University or college degree	59 564 (35.0)	57 082 (35.4)	2 482 (27.6)
Other	110 805(65.0)	104 283(64.6)	6 522(72.4)
Distance to major roads, 1/km (mean [*SD*])	0.01 (0.02)	0.01 (0.02)	0.01 (0.02)
24-h weighted average noise, dB (mean [*SD*])	55.94 (4.17)	55.94 (4.16)	55.98 (4.23)
Drinking frequency per week (mean [*SD*])	10.36 (9.90)	10.36 (9.83)	10.24 (11.13)
Smoking frequency per day (mean [*SD*])	6.41 (10.49)	6.30 (10.41)	8.21 (11.75)
Household income			
Low	76 198 (44.7)	70 851 (43.9)	5 347 (59.4)
Moderate	47 096 (27.6)	44 941 (27.9)	2 155 (23.9)
High	47 075 (27.6)	45 573 (28.2)	1 502 (16.7)
Social deprivation			
Low	57 062 (33.5)	54 476 (33.8)	2 586 (28.7)
Moderate	56 921 (33.4)	54 082 (33.5)	2 840 (31.5)
High	56 386 (33.1)	52 808 (32.7)	3 578 (39.7)
Genetic risk of MD			
Low	56 790 (33.3)	54 270 (33.6)	2 520 (28.0)
Moderate	56 789 (33.3)	53 825 (33.4)	2 965 (32.9)
High	56 790 (33.3)	53 271 (33.0)	3 519 (39.1)
NO_2_, μg/m^3^ (median [IQR])	25.34 (20.77–30.23)	25.3 (20.73–30.2)	26.1 (21.54–30.75)
NO, μg/m^3^ (median [IQR])	15.54 (11.24–20.02)	15.5 (11.20–19.99)	16.17 (11.89–20.6)
PM_2.5_, μg/m^3^ (median [IQR])	9.85 (9.21–10.46)	9.85 (9.20–10.45)	9.96 (9.33–10.58)
PM_2.5–10_, μg/m^3^ (median [IQR])	6.08 (5.83–6.58)	6.08 (5.83–6.58)	6.08 (5.84–6.6)

### Association between air pollutants and MDs

**Figure 2.**
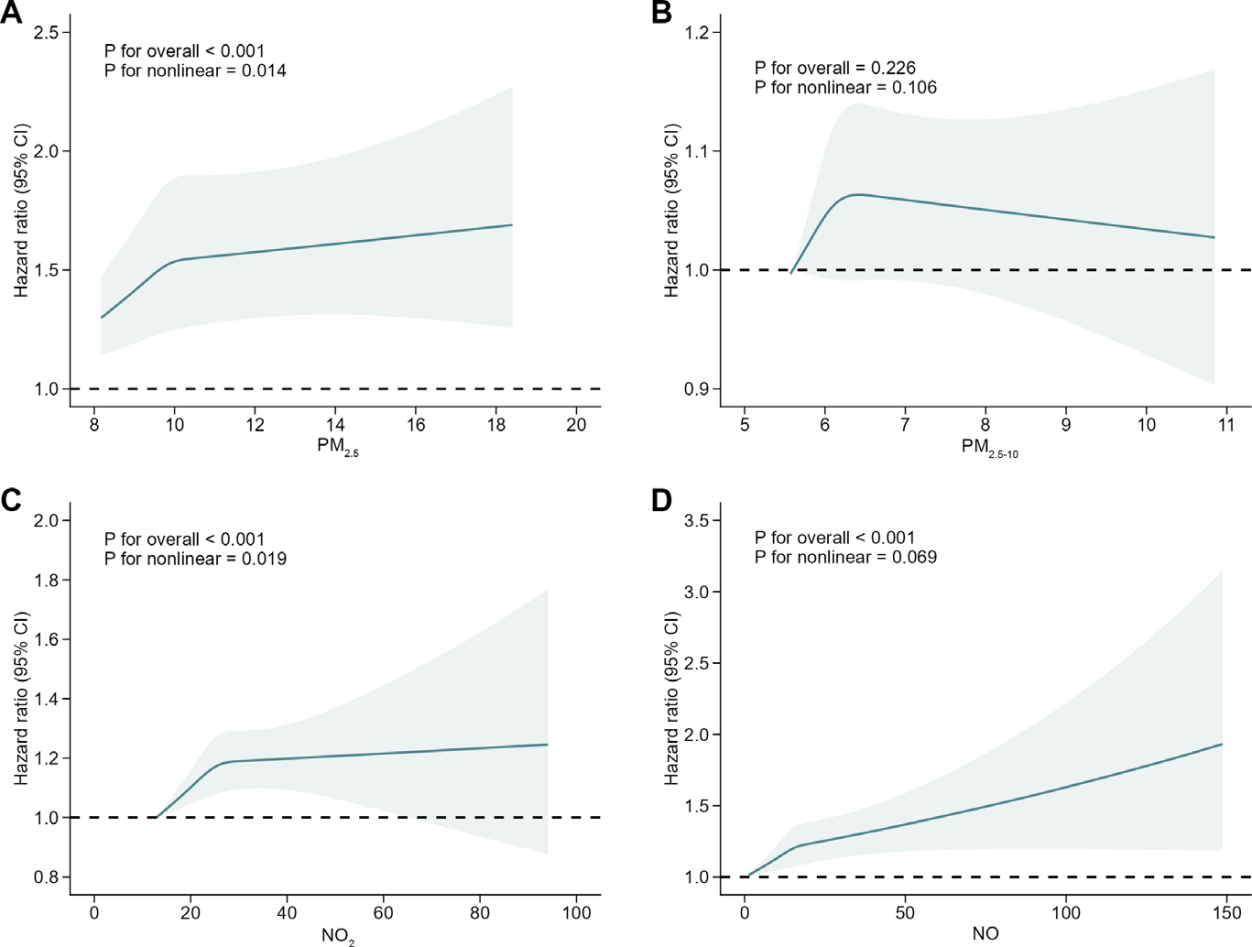
Dose–response curves between air pollutants and risk of mental disorders (MD).The models were adjusted for age, gender, education attainment, employment status, smoking frequency per day, drinking frequency per week, 24-h weighted average noise, distance to major roads, social deprivation, household income, and genetic risk of MD. PM_2.5_, particulate matter with aerodynamic diameter ≤ 2.5 μm; PM_2.5–10_, 2.5 μm < particulate matter with aerodynamic diameter ≤ 10 μm; NO_2_, nitrogen dioxide; NO, nitric oxide.

The associations of air pollutants and the risk of MD are shown in Table 2. In the main models, compared with the lowest quartile of NO_2_ concentration, the hazard ratios (HRs) of MD in quartiles 2–4 were 1.04 (95% CI, 0.98–1.11; *P* = 0.220), 1.11 (95% CI, 1.04–1.18; *P* = 0.001), and 1.12 (95% CI, 1.05–1.19; *P* = 7.73 × 10^−4^). For NO, the HRs of MD were 1.08 (95% CI, 1.02–1.15; *P* = 0.014), 1.11 (95% CI, 1.04–1.18; *P* = 8.13 × 10^−4^), and 1.10 (95% CI, 1.03–1.17; *P* = 0.004) in quartiles 2–4 compared with the lowest quartile. For PM_2.5_, the HRs for MD were 1.08 (95% CI, 1.02–1.15; *P* = 0.012), 1.09 (95% CI, 1.03–1.16; *P* = 0.005), and 1.16 (95% CI, 1.09–1.23; *P* = 6.56 × 10^−6^) in quartiles 2–4, respectively, as compared to the lowest quartile. The HRs of MD in quartiles 2–4 of PM_2.5–10_ were 1.11 (95% CI, 1.05–1.18; *P* = 5.89 × 10^−4^), 1.04 (95% CI, 0.98–1.10; *P* = 0.216), and 1.06 (95% CI, 1.00–1.13; *P* = 0.054) compared to the lowest quartile. The dose–response curves showed significant nonlinear association between PM_2.5_/NO_2_ with the incidence of MD, with steeper slopes at lower concentration and plateauing trends at higher exposure. We observed that the risk of MD increased as NO concentration increased but did not show a nonlinear trend (Figure 2).

**Table 2.** The hazard ratio (HR) with 95% confidence intervals (CI) of air pollutants for mental disorders (MDs)

	Quartile 1	Quartile 2		Quartile 3		Quartile 4	
		HR (95% CI)	*P* value	HR (95% CI)	*P* value	HR (95% CI)	*P* value
NO_2_							
Model 1	Ref.	1.12 (1.05–1.19)	2.37 × 10^−4^	1.24 (1.17–1.32)	6.66 × 10^−13^	1.31 (1.23–1.39)	1.18 × 10^−18^
Model 2	Ref.	1.09 (1.03–1.16)	0.005	1.21 (1.14–1.28)	8.33 × 10^−10^	1.30 (1.22–1.38)	2.88 × 10^−17^
Model 3	Ref.	1.04 (0.98–1.11)	0.220	1.11 (1.04–1.18)	0.001	1.12 (1.05–1.19)	7.73 × 10^−4^
NO							
Model 1	Ref.	1.15 (1.08–1.22)	1.11 × 10^−5^	1.23 (1.16–1.3)	1.90 × 10^−11^	1.29 (1.21–1.37)	7.95 × 10^−17^
Model 2	Ref.	1.13 (1.06–1.2)	6.82 × 10^−5^	1.2 (1.13–1.28)	1.25 × 10^−9^	1.27 (1.19–1.35)	1.02 × 10^−13^
Model 3	Ref.	1.08 (1.02–1.15)	0.014	1.11 (1.04–1.18)	8.13 × 10^−4^	1.10 (1.03–1.17)	0.004
PM_2.5_							
Model 1	Ref.	1.15 (1.08–1.22)	8.18 × 10^−6^	1.22 (1.15–1.30)	6.00 × 10^−11^	1.37 (1.29–1.45)	4.23 × 10^−25^
Model 2	Ref.	1.13 (1.07–1.21)	5.91 × 10^−5^	1.20 (1.12–1.27)	7.71 × 10^−9^	1.35 (1.27–1.43)	6.72 × 10^−22^
Model 3	Ref.	1.08 (1.02–1.15)	0.012	1.09 (1.03–1.16)	0.005	1.16 (1.09–1.23)	6.56 × 10^−6^
PM_2.5–10_							
Model 1	Ref.	1.15 (1.09–1.22)	1.66 × 10^−6^	1.10 (1.04–1.17)	0.002	1.12 (1.06–1.19)	1.75 × 10^−4^
Model 2	Ref.	1.14 (1.07–1.21)	1.17 × 10^−5^	1.09 (1.03–1.16)	0.005	1.10 (1.03–1.17)	0.002
Model 3	Ref.	1.11 (1.05–1.18)	5.89 × 10^−4^	1.04 (0.98–1.10)	0.216	1.06 (1.00–1.13)	0.054

*Note:* The air pollutant concentrations are expressed in quartiles. Model 1: adjusted for age, gender; Model 2: adjusted for age, gender, education attainment, employment status, smoking frequency per day, drinking frequency per week, 24-h weighted average noise, and distance to major roads; Model 3: adjusted for age, gender, education attainment, employment status, smoking frequency per day, drinking frequency per week, 24-h weighted average noise, distance to major roads, social deprivation, household income, and genetic risk of MD.

In the sensitivity analysis, we further adjusted healthy diet score and IPAQ activity based on the main model and found the robust associations between air pollution exposure and MD. For instance, the HR of PM_2.5_ in quartile 4 was 1.10 (95% CI, 1.04–1.19; *P* = 0.004); the HR of NO_2_ in quartile 4 was 1.09 (95% CI, 1.01–1.17; *P* = 0.023); the HR of NO in quartile 4 was 1.09 (95% CI, 1.01–1.17; *P* = 0.020); and the HR of PM_2.5–10_ in quartile 2 was 1.10 (95% CI, 1.03–1.17; *P* = 0.006), compared to lowest corresponding air pollutant concentration (Table S1). We also conducted analyses for single MDs and found that air pollutants were associated with various MDs, which were generally consistent with the results for major MD (Table S2–S5).

### Impact of socioeconomic status on the associations between air pollutants and MDs

Among individuals within different household income subgroups, PM_2.5_ was observed to increase the risk of MD (all *P*-trend < 0.05; Table S6). The risk of increased NO_2_ concentration for MD was only detected in low-income individuals. Furthermore, we observed that NO increases the risk of MD in low- and moderate-income individuals. In the high-income subgroup, apart from PM_2.5_, no significant associations were observed between other air pollutants and the risk of MD.

In each deprivation subgroup, PM_2.5_ exposure was found to be associated with an increased risk of MD (all *P*-trend < 0.05; Table S7). The HR of quartile 4 of PM_2.5_ concentration for MD was similar across different deprivation levels. The risk of NO_2_ exposure on MD was detected in individuals within low and moderate deprivation subgroups. For NO exposure, a heightened risk of MD was specifically identified within the moderate deprivation subgroup. There was no significant difference in the HR of PM_2.5–10_ concentrations for MD among individuals with low and high deprivation levels. Furthermore, we did not find significant interactions between different socioeconomic status and air pollutants.

### Impact of genetic risk on the associations between air pollutants and MDs

We observed that exposure to NO was associated with an increased risk of major MD only in individuals with high genetic risk (Table S8). In both high and low genetic risk individuals, exposure to PM_2.5_ and PM_2.5–10_ was associated with an increased risk of MD. Moreover, exposure to NO_2_ was observed to increase the risk of major MD across all genetic risk groups. We did not find significant interactions between MD genetic risk and air pollutants.

### Joint effect of socioeconomic status or genetic risk and air pollutants on the risk of MDs

**Figure 3.**
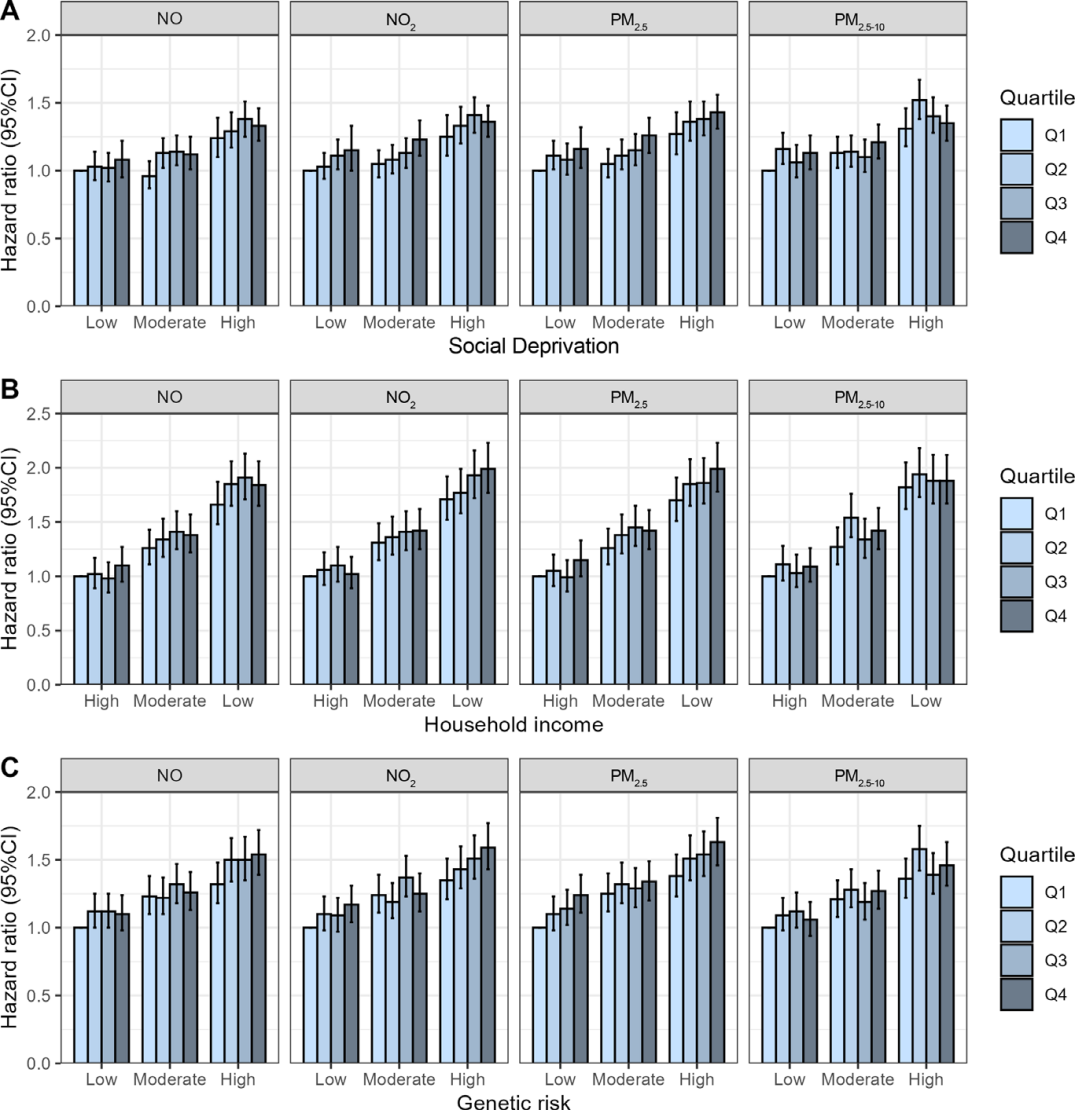
Joint effects of socioeconomic status or genetic risk and air pollutants on the risk of mental disorders (MD). Data were presented as hazard ratios with 95% confidence intervals for MD, adjusted for age, gender, education attainment, employment status, smoking frequency per day, drinking frequency per week, 24-h weighted average noise, and distance to major roads. The social deprivation, household income, and genetic risk of MD were adjusted appropriately. Participants with lowest air pollutants and highest socioeconomic status or lowest genetic risk were used as the reference group. PM_2.5_, particulate matter with aerodynamic diameter ≤ 2.5 μm; PM_2.5–10_, 2.5 μm < particulate matter with aerodynamic diameter ≤ 10 μm; NO_2_, nitrogen dioxide; NO, nitric oxide.

We observed significant effects of combined air pollutants and socioeconomic status on the risk of MD (Figure 3, Table S9–S10). The higher MD risk was detected in individuals with the lower socioeconomic status and higher air pollutants exposures. For example, compared to individuals with the highest household income and the lowest quartile of PM_2.5_, the HRs of MD in individuals with the lowest household income and highest quartile of PM_2.5_ were 1.99 (95% CI, 1.78–2.23). Compared to lowest deprivation and lowest quartile of NO_2_, highest deprivation and highest quartile of NO_2_ significantly increased the risk of MD (HR: 1.36, 95% CI, 1.25–1.48).

We also detected joint effects of air pollutants and genetic susceptibility on the risk of MD (Table S11). Compared to lowest genetic risk of MD and lowest concentration of air pollutants, highest genetic risk and highest quartile of air pollutants were significantly associated with the increased risk of MD (PM_2.5_: HR, 1.63, 95% CI, 1.46–1.81; PM_2.5–10_: 1.46, 1.31–1.63; NO_2_: 1.59, 1.43–1.77; and NO: 1.54, 1.39–1.72). Moreover, we found significant joint effects of a high genetic risk for MD and low concentrations of air pollutants. For instance, compared with lowest genetic risk and lowest concentrations of PM_2.5_, highest genetic risk for MD and lowest concentrations of PM_2.5_ was significantly associated with the risk of MD (HR: 1.38, 95% CI: 1.23–1.54); compared with lowest genetic risk and lowest concentrations of NO_2_, highest genetic risk for MD and lowest concentrations of NO_2_ significantly increased the risk of MD (HR: 1.35, 95% CI: 1.21–1.51).

## Discussion

The damage of air pollutants to mental health has gradually been recognized. Given the shared neural basis of psychiatric disorders and the high prevalence of psychiatric comorbidities [29], exploring the impact of long-term air pollution exposure on overall MD has important implications for mental health. Based on the UK Biobank, we selected depression, anxiety, bipolar disorder, and schizophrenia as the main MD for a measure of overall mental impairment. We found that long-term air pollution exposure (PM_2.5_, PM_2.5–10_, NO_2_, and NO) significantly increased the risk of MD. Meanwhile, socioeconomic status and genetic susceptibility were found to have significantly effect on the association between air pollution and MD.

Exposure to air pollutants has been demonstrated to significantly elevate the risk of various mental health disorders, including depression [6], anxiety [6], bipolar disorder [30], and schizophrenia [31], each to varying degrees. Acute exposure to PM_10_ and PM_2.5_ may contribute to a substantial deterioration in mental well-being [32]. A comprehensive investigation into the short-term effects of air pollution on the incidence of MD across three Chinese cities revealed a noteworthy increase in daily outpatient visits for mental health issues, with a particularly pronounced impact linked to NO_2_ [33]. In addition, increases in the IQRs of NO_2_, NO_X_, and PM_2.5_ were found to be significantly associated with prolonged in-patient days for psychotic and mood disorders after 1 year [34]. Environmental-Risk Longitudinal Twin Study reported a substantial association with the emergence of psychotic experiences during adolescence, especially outdoor exposure to NO_2_, NO_X_, and PM [21]. Furthermore, the dose–response relationship showed steeper curves at low air pollution and flattened out with increasing concentrations, indicating that the risk of developing MD tends to increase more rapidly at lower levels of air pollution, leveling off as exposure levels rise. This finding carries significant policy implications for air pollution control. Consistent with previous studies, the risk of MD remained significant despite air pollutant concentrations below the World Health Organization (WHO) air quality guidelines limits [21].

Air pollution may potentially induce structural and functional changes in the CNS related to psychiatric disorders by mediating systemic inflammation and cerebral oxidative stress [35]. It is reported that air pollutants can penetrate lung tissue compartments and enter the circulatory system to the brain, where they can activate microglial activity, leading to neuroinflammation and increased production of intracellular reactive oxygen species, thereby causing neuronal apoptosis [35-38]. The hypothalamic–pituitary–adrenal (HPA) axis plays a pivotal role in the pathophysiology of MD. PM_2.5_ may elevate the risk of MD by impacting the normal functioning of the HPA axis, disrupting the balance of the neuroendocrine system [39]. Mice exposed to PM_2.5_ showed reduced expression of hippocampal glucocorticoid receptors and increased levels of glucocorticoids, potentially maintaining the HPA axis in an activated state [39]. Additionally, air pollutants’ exposure was found to be associated with hippocampal atrophy in individuals experiencing their first episode of schizophrenia [40]. Olfactory bulb (OB) damage is frequently observed in patients with anxiety disorders, depression, schizophrenia, and bipolar disorder [41]. A recent study observed that the OB of mice exposed to PM_2.5_ exhibited reductions in tissue cells, activation of microglial cells, and enrichment of tumor necrosis factor-mediated signaling [42]. In early adolescence, higher NO_2_ exposure has been linked to reduced functional connectivity from cortical networks to subcortical networks [43]. Acute NO_2_ inhalation can result in brain damage through mitochondrial disruption [38].

We observed significant associations between NO_2_ and NO and the risk of MD among individuals within low-income subgroup. Previous study noted a significant correlation between exposure to NO_2_ and depression symptoms in low-income individuals, while not in individuals with high income [13]. We also found that PM_2.5_ had a significant impact on the risk of MD across all income subgroups, with the highest risk observed in the high-income population. Previous research also revealed that individuals with higher family incomes are more susceptible to an increased risk of depression and anxiety due to PM_2.5_ exposure [44]. Therefore, it is essential to pay attention to the risk of PM_2.5_ for MD, even in high-income individuals. We did not observe substantial differences in the impact of air pollutants on MD risk across different deprivation levels, which is consistent with a previous study on air pollution and Index of Multiple Deprivation (IMD) [34]. However, concerning household income and social deprivation, we did observe that their combined effects with air pollution significantly influenced MDs. Previous studies have established that individuals with lower socioeconomic status are at a higher risk of developing MDs [11]. Individuals suffering from depression or anxiety disorders are more likely to reside in communities characterized by lower socioeconomic status and higher levels of air pollution [45]. The stress associated with economic disadvantage may contribute to a higher risk of developing or exacerbating psychological problems [46]. Community social capital might act as a buffer between environmental hazards and residents’ mental well-being [47]. In summary, participants with lower socioeconomic status are more likely to experience MD when exposed to high levels of air pollution. Preventing the accumulation of adverse factors and intervening with individuals who are more susceptible to the negative effects of air pollution is of paramount importance.

This study has some strengths. First, this prospective study evaluated the impact of long-term exposure to air pollution on the risk of major MD, which has large sample size and helps assess the impact of air pollution as a general psychopathological factor. Additionally, our investigation delved into the relationship between air pollution and MD across diverse socioeconomic status as well as genetic susceptibility, providing insights for the development of targeted interventions for individuals with genetic vulnerability and socioeconomic disadvantage. Our findings carry significant implications for mitigating the mental health burden associated with air pollution. We observed that air pollution, as a general psychopathological factor, may still increase the risk of MD even at levels below the WHO air quality guideline limits. This underscores the need to establish stricter air quality standards and further reduce the emissions of PM_2.5_ and other pollutants. Additionally, preventing the cumulative effects of air pollution exposure alongside other adverse factors is critical. Public health interventions should integrate individual socioeconomic status and genetic risk levels to design personalized risk management strategies that minimize the accumulation of harmful factors. Moreover, targeted interventions should be prioritized for individuals who are more vulnerable to the negative impacts of air pollution. Efforts should focus on protecting the health of socioeconomically disadvantaged populations. This includes improving the living environments of low-income individuals and enhancing access to mental health services, ensuring these vulnerable groups receive adequate support and care.

Several limitations should be noted. First, this study only included white British individuals, so our findings should be applied with caution to other populations. Second, we used data at baseline to measure air pollution exposure, which may not reflect trends during follow-up. According to previous study, the time trend fluctuations for most air pollution were generally stable during the UK Biobank follow-up period [48]. It has been shown that using annual mean air pollutants at the baseline and using time-varying air pollutants results in overall similar results [49]. Moreover, although we conducted separate analyses for each MD, the limited sample sizes for schizophrenia and bipolar disorder may have reduced the statistical power. Additionally, MDs often overlap, which might contribute to some degree of data distortion when aggregating multiple disorders in the major MD analysis. Moreover, while our findings highlight the general neurotoxic effects of air pollution on mental health, the heterogeneity among different MDs suggests that the underlying mechanisms and susceptibilities may vary. These limitations underscore the need for further studies with larger sample sizes and detailed mechanistic investigations to validate and expand upon our findings. Furthermore, potential confounding factors may have affected our findings. Thus, we performed a sensitivity analysis with additional adjustments for diet and exercise. The sensitivity analysis confirmed the robustness of our results. However, it is important to acknowledge that unknown or unmeasured factors may still contribute to residual confounding.

In conclusion, this large-scale prospective cohort study revealed significant associations between long-term exposure to ambient air pollution and an elevated risk of major MD. Individuals with low household income appeared more vulnerable to the adverse effects of air pollution on mental health. Moreover, the combined impact of air pollution with low socioeconomic status and high genetic susceptibility significantly amplified the risk of major MD. These findings highlight the importance of air pollution control in alleviating the mental health burdens, particularly among socioeconomically disadvantaged individuals and those with high genetic predisposition.

## Supporting information

Pan et al. supplementary material Pan et al. supplementary material

## Data Availability

The UKB data support the findings of this study are openly available in the UK Biobank Access Management System at https://www.ukbiobank.ac.uk/.

## Supplementary material

The supplementary material for this article can be found at http://doi.org/10.1192/j.eurpsy.2024.1809.

## References

[1] Collaborators GMD. Global, regional, and national burden of 12 mental disorders in 204 countries and territories, 1990-2019: a systematic analysis for the Global Burden of Disease Study 2019. Lancet Psychiatry. 2022;9(2):137–50. 10.1016/s2215-0366(21)00395-3. 35026139 PMC8776563

[2] Christensen MK, Lim CCW, Saha S, Plana-Ripoll O, Cannon D, Presley F, et al. The cost of mental disorders: a systematic review. Epidemiol Psychiatr Sci. 2020;29:e161. 10.1017/s204579602000075x. 32807256 PMC7443800

[3] Collaborators GRF. Global burden of 87 risk factors in 204 countries and territories, 1990-2019: a systematic analysis for the Global Burden of Disease Study 2019. Lancet. 2020;396(10258):1223–49. 10.1016/s0140-6736(20)30752-2. 33069327 PMC7566194

[4] Buoli M, Grassi S, Caldiroli A, Carnevali GS, Mucci F, Iodice S, et al. Is there a link between air pollution and mental disorders? Environ Int. 2018;118:154–68. 10.1016/j.envint.2018.05.044. 29883762

[5] Wu M, Xie J, Zhou Z, Wang L, Hu Y, Sun Y, et al. Fine particulate matter, vitamin D, physical activity, and major depressive disorder in elderly adults: results from UK Biobank. J Affect Disord. 2022;299:233–8. 10.1016/j.jad.2021.12.009. 34879260

[6] Gao X, Jiang M, Huang N, Guo X, Huang T. Long-term air pollution, genetic susceptibility, and the risk of depression and anxiety: a prospective study in the UK Biobank cohort. Environ Health Perspect. 2023;131(1):17002. 10.1289/ehp10391. 36598457 PMC9812022

[7] Caspi A, Houts RM, Belsky DW, Goldman-Mellor SJ, Harrington H, Israel S, et al. The p factor: one general psychopathology factor in the structure of psychiatric disorders? Clin Psychol Sci. 2014;2(2):119–37. 10.1177/2167702613497473. 25360393 PMC4209412

[8] Hansen JY, Shafiei G, Vogel JW, Smart K, Bearden CE, Hoogman M, et al. Local molecular and global connectomic contributions to cross-disorder cortical abnormalities. Nat Commun. 2022;13(1):4682. 10.1038/s41467-022-32420-y. 35948562 PMC9365855

[9] Paksarian D, Trabjerg BB, Merikangas KR, Mors O, Børglum AD, Hougaard DM, et al. Adolescent residential mobility, genetic liability and risk of schizophrenia, bipolar disorder and major depression. Br J Psychiatry. 2020;217(1):390–6. 10.1192/bjp.2020.8. 32024557 PMC8130005

[10] Jbaily A, Zhou X, Liu J, Lee TH, Kamareddine L, Verguet S, et al. Air pollution exposure disparities across US population and income groups. Nature. 2022;601(7892):228–33. 10.1038/s41586-021-04190-y. 35022594 PMC10516300

[11] Hakulinen C, Komulainen K, Suokas K, Pirkola S, Pulkki-Råback L, Lumme S, et al. Socioeconomic position at the age of 30 and the later risk of a mental disorder: a nationwide population-based register study. J Epidemiol Community Health. 2023;77(5):298–304. 10.1136/jech-2022-219674. 36746629 PMC10086472

[12] Barnett K, Mercer SW, Norbury M, Watt G, Wyke S, Guthrie B. Epidemiology of multimorbidity and implications for health care, research, and medical education: a cross-sectional study. Lancet. 2012;380(9836):37–43. 10.1016/s0140-6736(12)60240-2. 22579043

[13] Zare Sakhvidi MJ, Lafontaine A, Lequy E, Berr C, de Hoogh K, Vienneau D, et al. Ambient air pollution exposure and depressive symptoms: findings from the French CONSTANCES cohort. Environ Int. 2022;170:107622. 10.1016/j.envint.2022.107622. 36384066

[14] Smoller JW, Andreassen OA, Edenberg HJ, Faraone SV, Glatt SJ, Kendler KS. Psychiatric genetics and the structure of psychopathology. Mol Psychiatry. 2019;24(3):409–20. 10.1038/s41380-017-0010-4. 29317742 PMC6684352

[15] Li D, Xie J, Wang L, Sun Y, Hu Y, Tian Y. Genetic susceptibility and lifestyle modify the association of long-term air pollution exposure on major depressive disorder: a prospective study in UK Biobank. BMC Med. 2023;21(1):67. 10.1186/s12916-023-02783-0. 36810050 PMC9945634

[16] Consortium C-DGotPG. Genomic relationships, novel loci, and pleiotropic mechanisms across eight psychiatric disorders. Cell. 2019;179(7):1469–82.e11. 10.1016/j.cell.2019.11.020. 31835028 PMC7077032

[17] Zhai S, Guo B, Wu B, Mehrotra DV, Shen J. Integrating multiple traits for improving polygenic risk prediction in disease and pharmacogenomics GWAS. Brief Bioinform. 2023. 10.1093/bib/bbad181. 37200155

[18] Sudlow C, Gallacher J, Allen N, Beral V, Burton P, Danesh J, et al. UK Biobank: an open access resource for identifying the causes of a wide range of complex diseases of middle and old age. PLoS Med. 2015;12(3):e1001779. 10.1371/journal.pmed.1001779. 25826379 PMC4380465

[19] Beelen R, Hoek G, Vienneau D, Eeftens M, Dimakopoulou K, Pedeli X, et al. Development of NO2 and NOx land use regression models for estimating air pollution exposure in 36 study areas in Europe – the ESCAPE project. Atmospheric Environment. 2013;72:10–23. 10.1016/j.atmosenv.2013.02.037.

[20] Eeftens M, Beelen R, de Hoogh K, Bellander T, Cesaroni G, Cirach M, et al. Development of land use regression models for PM(2.5), PM(2.5) absorbance, PM(10) and PM(coarse) in 20 European study areas: results of the ESCAPE project. Environ Sci Technol. 2012;46(20):11195–205. 10.1021/es301948k. 22963366

[21] Newbury JB, Arseneault L, Beevers S, Kitwiroon N, Roberts S, Pariante CM, et al. Association of air pollution exposure with psychotic experiences during adolescence. JAMA Psychiatry. 2019;76(6):614–23. 10.1001/jamapsychiatry.2019.0056. 30916743 PMC6499472

[22] Jiménez M, Abad FJ, Garcia-Garzon E, Golino H, Christensen AP, Garrido LE. Dimensionality assessment in bifactor structures with multiple general factors: a network psychometrics approach. Psychol Methods. 2023. 10.1037/met0000590. 37410419

[23] Abdi H, Williams LJ. Principal component analysis. Wiley Interdiscip Rev Comput Stat. 2010;2(4):433–59. 10.1002/wics.101.

[24] Choi SW, O’Reilly PF. PRSice-2: polygenic risk score software for biobank-scale data. Gigascience. 2019;8(7):1–6. 10.1093/gigascience/giz082. PMC6629542 31307061

[25] Karlsson Linnér R, Biroli P, Kong E, Meddens SFW, Wedow R, Fontana MA, et al. Genome-wide association analyses of risk tolerance and risky behaviors in over 1 million individuals identify hundreds of loci and shared genetic influences. Nat Genet. 2019;51(2):245–57. 10.1038/s41588-018-0309-3. 30643258 PMC6713272

[26] Mozaffarian D. Dietary and policy priorities for cardiovascular disease, diabetes, and obesity: a comprehensive review. Circulation. 2016;133(2):187–225. 10.1161/circulationaha.115.018585. 26746178 PMC4814348

[27] Lourida I, Hannon E, Littlejohns TJ, Langa KM, Hyppönen E, Kuzma E, et al. Association of lifestyle and genetic risk with incidence of dementia. JAMA. 2019;322(5):430–7. 10.1001/jama.2019.9879. 31302669 PMC6628594

[28] Committee IR. Guidelines for data processing and analysis of the International Physical Activity Questionnaire (IPAQ)-short and long forms. http://www.ipaq ki se/scoring pdf. 2005.

[29] Xie C, Xiang S, Shen C, Peng X, Kang J, Li Y, et al. A shared neural basis underlying psychiatric comorbidity. Nat Med. 2023;29(5):1232–42. 10.1038/s41591-023-02317-4. 37095248 PMC10202801

[30] Li D, Ma Y, Cui F, Yang Y, Liu R, Tang L, et al. Long-term exposure to ambient air pollution, genetic susceptibility, and the incidence of bipolar disorder: a prospective cohort study. Psychiatry Res. 2023;327:115396. 10.1016/j.psychres.2023.115396. 37549511

[31] Song R, Liu L, Wei N, Li X, Liu J, Yuan J, et al. Short-term exposure to air pollution is an emerging but neglected risk factor for schizophrenia: a systematic review and meta-analysis. Sci Total Environ. 2022;1(854):158823. 10.1016/j.scitotenv.2022.158823. 36116638

[32] Muhsin HA, Steingrimsson S, Oudin A, Åström DO, Carlsen HK. Air pollution and increased number of psychiatric emergency room visits: a case-crossover study for identifying susceptible groups. Environ Res. 2022;204 (Pt A):112001. 10.1016/j.envres.2021.112001. 34499892

[33] Li H, Zhang S, Qian ZM, Xie XH, Luo Y, Han R, et al. Short-term effects of air pollution on cause-specific mental disorders in three subtropical Chinese cities. Environ Res. 2020;191:110214. 10.1016/j.envres.2020.110214. 32946889

[34] Newbury JB, Stewart R, Fisher HL, Beevers S, Dajnak D, Broadbent M, et al. Association between air pollution exposure and mental health service use among individuals with first presentations of psychotic and mood disorders: retrospective cohort study. Br J Psychiatry. 2021;219(6):678–85. 10.1192/bjp.2021.119. 35048872 PMC8636613

[35] Block ML, Calderón-Garcidueñas L. Air pollution: mechanisms of neuroinflammation and CNS disease. Trends Neurosci. 2009;32(9):506–16. 10.1016/j.tins.2009.05.009. 19716187 PMC2743793

[36] Thiankhaw K, Chattipakorn N, Chattipakorn SC. PM2.5 exposure in association with AD-related neuropathology and cognitive outcomes. Environ Pollut. 2022;292 (Pt A):118320. 10.1016/j.envpol.2021.118320. 34634399

[37] Ehsanifar M, Montazeri Z, Zavareh MS, Rafati M, Wang J. Cognitive impairment, depressive-like behaviors and hippocampal microglia activation following exposure to air pollution nanoparticles. Environ Sci Pollut Res Int. 2023;30(9):23527–37. 10.1007/s11356-022-23882-0. 36327074

[38] Yan W, Ji X, Shi J, Li G, Sang N. Acute nitrogen dioxide inhalation induces mitochondrial dysfunction in rat brain. Environ Res. 2015;138:416–24. 10.1016/j.envres.2015.02.022. 25791864

[39] Jia Z, Wei Y, Li X, Yang L, Liu H, Guo C, et al. Exposure to ambient air particles increases the risk of mental disorder: Findings from a natural experiment in Beijing. Int J Environ Res Public Health. 2018;15(1):160. 10.3390/ijerph15010160. 29351245 PMC5800259

[40] Worthington MA, Petkova E, Freudenreich O, Cather C, Holt D, Bello I, et al. Air pollution and hippocampal atrophy in first episode schizophrenia. Schizophr Res. 2020;218:63–9. 10.1016/j.schres.2020.03.001. 32169403

[41] Marin C, Alobid I, Fuentes M, López-Chacón M, Mullol J. Olfactory dysfunction in mental illness. Curr Allergy Asthma Rep. 2023;23(3):153–64. 10.1007/s11882-023-01068-z. 36696016 PMC9875195

[42] Ji X, Liu R, Guo J, Li Y, Cheng W, Pang Y, et al. Olfactory bulb microglia activation mediated neuronal death in real-ambient particulate matter exposure mice with depression-like behaviors. Sci Total Environ. 2022;821:153456. 10.1016/j.scitotenv.2022.153456. 35093369

[43] Cotter DL, Campbell CE, Sukumaran K, McConnell R, Berhane K, Schwartz J, et al. Effects of ambient fine particulates, nitrogen dioxide, and ozone on maturation of functional brain networks across early adolescence. Environ Int. 2023;177:108001. 10.1016/j.envint.2023.108001. 37307604 PMC10353545

[44] Pun VC, Manjourides J, Suh H. Association of ambient air pollution with depressive and anxiety symptoms in older adults: results from the NSHAP study. Environ Health Perspect. 2017;125(3):342–8. 10.1289/ehp494. 27517877 PMC5332196

[45] Generaal E, Timmermans EJ, Dekkers JEC, Smit JH, Penninx B. Not urbanization level but socioeconomic, physical and social neighbourhood characteristics are associated with presence and severity of depressive and anxiety disorders. Psychol Med. 2019;49(1):149–61. 10.1017/s0033291718000612. 29540253 PMC6316373

[46] Qiu X, Wei Y, Weisskopf M, Spiro A, Shi L, Castro E, et al. Air pollution, climate conditions and risk of hospital admissions for psychotic disorders in U.S. residents. Environ Res. 2023;216(Pt 2):114636. 10.1016/j.envres.2022.114636. 36283440 PMC9712244

[47] Ard K, Colen C, Becerra M, Velez T. Two mechanisms: the role of social capital and industrial pollution exposure in explaining racial disparities in self-rated health. Int J Environ Res Public Health. 2016;13(10). 10.3390/ijerph13101025. PMC5086764 27775582

[48] Furlong MA, Klimentidis YC. Associations of air pollution with obesity and body fat percentage, and modification by polygenic risk score for BMI in the UK Biobank. Environ Res. 2020;185:109364. 10.1016/j.envres.2020.109364. 32247148 PMC7199644

[49] Wong CM, Lai HK, Tsang H, Thach TQ, Thomas GN, Lam KB, et al. Satellite-based estimates of long-term exposure to fine particles and association with mortality in elderly Hong Kong residents. Environ Health Perspect. 2015;123(11):1167–72. 10.1289/ehp.1408264. 25910279 PMC4629733

