# Supplementary material for: Long-term ambient air pollution and the risk of major mental disorder: A prospective cohort study
Source: Eur Psychiatry. 2024 Dec 18;68(1):e1. doi: 10.1192/j.eurpsy.2024.1809 (PMC11823001; doi:10.1192/j.eurpsy.2024.1809)
Supplement: Pan et al. supplementary material [file S0924933824018091sup001.docx]

**Supplementary Materials**

**Long-term ambient air pollution and the risk of cross-mental disorder:**

**a prospective cohort study**

Chuyu Pan^*1^, Bolun Cheng^*1^, Shiqiang Cheng^1^, Li Liu^1^, Xuena Yang^1^, Peilin Meng^1^, Xin Qi^2^, Na Zhang^1^, Xiaoyue Qin^1^, Dan He^1^, Wenming Wei^1^, Jingni Hui^1^, Yan Wen^1^, Yumeng Jia^1^, Huan Liu^1^, Feng Zhang^#1^

^1^Key Laboratory of Trace Elements and Endemic Diseases of National Health and Family Planning Commission, School of Public Health, Health Science Center, Xi’an Jiaotong University, Xi’an, China

^2^Precision Medicine Center, The First Affiliated Hospital of Xi'an Jiaotong University, Xi'an, P. R. China.

^*^The first two authors contributed equally to the work

^#^Corresponding author:

Feng Zhang

Key Laboratory of Trace Elements and Endemic Diseases, National Health Commission of

the People’s Republic of China. School of Public Health, Health Science Center, Xi’an Jiaotong University

Email: [fzhxjtu@xjtu.edu.cn](mailto:fzhxjtu@xjtu.edu.cn)

Xi’an, P. R. China 710061

**Supplementary Methods**

***The detailed definition of mental disorders (MD)***

| Mental disorders | ICD10 codes |
| --- | --- |
| Depression | F32 Depressive episode  F32.0 Mild depressive episode  F32.1 Moderate depressive episode  F32.2 Severe depressive episode without psychotic symptoms  F32.3 Severe depressive episode with psychotic symptoms  F32.8 Other depressive episodes  F32.9 Depressive episode, unspecified  F33 Recurrent depressive disorder  F33.0 Recurrent depressive disorder, current episode mild  F33.1 Recurrent depressive disorder, current episode moderate  F33.2 Recurrent depressive disorder, current episode severe without psychotic symptoms  F33.3 Recurrent depressive disorder, current episode severe with psychotic symptoms  F33.4 Recurrent depressive disorder, currently in remission  F33.8 Other recurrent depressive disorders  F33.9 Recurrent depressive disorder, unspecified |
| Anxiety Disorders | F40 Phobic anxiety disorders  F40.0 Agoraphobia  F40.1 Social phobias  F40.2 Specific (isolated) phobias  F40.8 Other phobic anxiety disorders  F40.9 Phobic anxiety disorder, unspecified  F41 Other anxiety disorders  F41.0 Panic disorder [episodic paroxysmal anxiety]  F41.1 Generalised anxiety disorder  F41.2 Mixed anxiety and depressive disorder  F41.3 Other mixed anxiety disorders  F41.8 Other specified anxiety disorders  F41.9 Anxiety disorder, unspecified |
| Bipolar Disorders | F31.0 Bipolar affective disorder, current episode hypomanic  F31.1 Bipolar affective disorder, current episode manic without psychotic symptoms  F31.2 Bipolar affective disorder, current episode manic with psychotic symptoms  F31.3 Bipolar affective disorder, current episode mild or moderate depression  F31.4 Bipolar affective disorder, current episode severe depression without psychotic symptoms  F31.5 Bipolar affective disorder, current episode severe depression with psychotic symptoms  F31.6 Bipolar affective disorder, current episode mixed  F31.7 Bipolar affective disorder, currently in remission  F31.8 Other bipolar affective disorders  F31.9 Bipolar affective disorder, unspecified |
| Schizophrenia | F20.0 Paranoid schizophrenia  F20.1 Hebephrenic schizophrenia  F20.2 Catatonic schizophrenia  F20.3 Undifferentiated schizophrenia  F20.4 Postschizophrenic depression  F20.5 Residual schizophrenia  F20.6 Simple schizophrenia  F20.8 Other schizophrenia  F20.9 Schizophrenia, unspecified  F21 Schizotypal disorder  F22 Persistent delusional disorders  F22.0 Delusional disorder  F22.8 Other persistent delusional disorders  F22.9 Persistent delusional disorder, unspecified  F23 Acute and transient psychotic disorders  F23.0 Acute polymorphic psychotic disorder without symptoms of schizophrenia  F23.1 Acute polymorphic psychotic disorder with symptoms of schizophrenia  F23.2 Acute schizophrenia-like psychotic disorder  F23.3 Other acute predominantly delusional psychotic disorders  F23.8 Other acute and transient psychotic disorders  F23.9 Acute and transient psychotic disorder, unspecified  F24 Induced delusional disorder  F25 Schizoaffective disorders  F25.0 Schizoaffective disorder, manic type  F25.1 Schizoaffective disorder, depressive type  F25.2 Schizoaffective disorder, mixed type  F25.8 Other schizoaffective disorders  F25.9 Schizoaffective disorder, unspecified  F28 Other nonorganic psychotic disorders  F29 Unspecified nonorganic psychosis |

***UK Biobank genotyping, imputation and quality control***

The genetic data from the UK Biobank included genotypes from 488,377 individuals assessed by two similar genotyping arrays: the Applied Biosystems UK BiLEVE Axiom Array and the Applied Biosystems UK Biobank Axiom[1]. IMPUTE4 was used for imputation, which was conducted in chunks of approximately 50 000 imputed markers with a 250 kb buffer region and on 5 000 samples per compute job. Routine quality control was performed during sample and DNA extraction, as well as genotyping. To ensure the consistency of genotype calling, statistical tests were performed to identify poor quality markers due to batch effects, plate effects, and deviation from Hardy-Weinberg equilibrium (HWE)[1]. Principal component analysis (PCA) was used to account for population structure in both sampl×10-based quality and marker control[1]. Additionally, individuals were restricted to only ‘white British’ based on self-reported ethnicity. KING software was applied to exclude genetically related individuals by performing kinship coefficient estimation[2].

***GWAS datasets for mental disorders (MD)***

The GWAS of MD were derived from Psychiatric Genomics Consortium (PGC) (<https://pgc.unc.edu/>), which is the largest biological investigation in the history of psychiatry. Briefly, the GWAS summary data of depression was derived from a large scale meta-analysis based in 135,458 cases and 344,901 controls[3]. Due to data limitations, we used data excluding individuals in 23andMe study and UK Biobank cohort. For anxiety disorders, we used data from GWAS for generalized anxiety disorders which conducting comparison between categorical AD cases and supernormal controls (N= 17,310)[4]. For bipolar disorders, the summary data was from a GWAS study of 41,917 bipolar disorder cases and 371,549 controls of European ancestry[5]. The GWAS summary for schizophrenia were derived from a GWAS for European ancestry containing 53,386 cases and 77,258 controls[6]. The detailed information on genotyping, imputation, and quality control can be found in the original study.

***Polygenic risk score (PRS)***

PRsic×10-2[7] was utilized to generate GRS for depression, anxiety disorders, bipolar disorders and schizophrenia. The clumping algorithm was set to identify any SNPs within 500 kb in LD with an r^2^ threshold greater than 0.2.[8] Age, gender and 10 principal components of population structure were used as covariates. We only included white British individuals and who were not genetically related. The best model was derived from testing the inclusion of SNPs with a range of *P* values in the dataset (5 ×10^-8^ to 1 interval of 5 ×10^-5^), to see which threshold gave the largest Nagelkerke’s R^2^ value to generate GRSs for each individual. We determined *P* value threshold of 0.02 for depression (Nagelkerke’s R^2^max = 1.04%), 0.06 for anxiety disorders (Nagelkerke’s R^2^max = 1.35%), 0.18 for schizophrenia (Nagelkerke’s R^2^max = 0.28%) and 1 for bipolar disorders (Nagelkerke’s R^2^max = 18.53%) were the best significance threshold for inclusion in this study.

***Linkage disequilibrium score (LDSC) regression***

LDSC regression analysis is a reliable and efficient method to identify the shared genetic architecture of human complex traits, estimating the heritability of diseases and testing their genetic correlation, primarily based on GWAS summary data.[9] We conducted LDSC to evaluate the genetic correlations across four mental disorders, and to construct a genetic correlation matrix. The European LD scores calculated from the 1000 Genomes by the developers were used as the linkage disequilibrium reference panel (https://github.com/bulik/ldsc).

***Healthy diet score***

The diet was assessed using a healthy diet score based on the Mediterranean diet and heart-healthy dietary recommendations for reducing the risk of chronic diseases [10, 11]. This score included seven components: fruits, vegetables, fish, processed meat, unprocessed red meat, whole grains, and refined grains. The healthy diet score was calculated by summing the scores of the seven food components consumed by each participant, ranging from 0 to 7 [11]. Higher scores indicating healthier dietary patterns.

***International Physical Activity Questionnaire (IPAQ)***

Exercise was evaluated using International Physical Activity Questionnaire (IPAQ), and participants were classified into three levels of physical activity groups: high, moderate and low[12]. The high IPAQ group was defined as engaging in at least one additional hour of moderat×10-intensity activity above basal levels daily, or at least half an hour of vigorous-intensity activity above basal levels daily. The moderate group was defined as doing some activity, roughly equivalent to 30 minutes of moderat×10-intensity physical activity on most days. The low IPAQ group included individuals who did not meet the criteria for either the high or moderate activity groups.

**Reference**

[1] Bycroft C, Freeman C, Petkova D, Band G, Elliott LT, Sharp K, et al. The UK Biobank resource with deep phenotyping and genomic data. Nature. 2018;562(7726):203-9. <https://doi.org/10.1038/s41586-018-0579-z>.

[2] Manichaikul A, Mychaleckyj JC, Rich SS, Daly K, Sale M, Chen WM. Robust relationship inference in genome-wide association studies. Bioinformatics. 2010;26(22):2867-73. <https://doi.org/10.1093/bioinformatics/btq559>.

[3] Wray NR, Ripke S, Mattheisen M, Trzaskowski M, Byrne EM, Abdellaoui A, et al. Genome-wide association analyses identify 44 risk variants and refine the genetic architecture of major depression. Nat Genet. 2018;50(5):668-81. <https://doi.org/10.1038/s41588-018-0090-3>.

[4] Otowa T, Hek K, Lee M, Byrne EM, Mirza SS, Nivard MG, et al. Meta-analysis of genome-wide association studies of anxiety disorders. Mol Psychiatry. 2016;21(10):1391-9. <https://doi.org/10.1038/mp.2015.197>.

[5] Mullins N, Forstner AJ, O'Connell KS, Coombes B, Coleman JRI, Qiao Z, et al. Genome-wide association study of more than 40,000 bipolar disorder cases provides new insights into the underlying biology. Nat Genet. 2021;53(6):817-29. <https://doi.org/10.1038/s41588-021-00857-4>.

[6] Trubetskoy V, Pardiñas AF, Qi T, Panagiotaropoulou G, Awasthi S, Bigdeli TB, et al. Mapping genomic loci implicates genes and synaptic biology in schizophrenia. Nature. 2022;604(7906):502-8. <https://doi.org/10.1038/s41586-022-04434-5>.

[7] Euesden J, Lewis CM, O’Reilly PF. PRSice: Polygenic Risk Score software. Bioinformatics. 2015;31(9):1466-8. <https://doi.org/10.1093/bioinformatics/btu848>.

[8] Piekos JA, Hellwege JN, Zhang Y, Torstenson ES, Jarvik GP, Dikilitas O, et al. Uterine fibroid polygenic risk score (PRS) associates and predicts risk for uterine fibroid. Hum Genet. 2022;141(11):1739-48. <https://doi.org/10.1007/s00439-022-02442-z>.

[9] Ni G, Moser G, Wray NR, Lee SH. Estimation of Genetic Correlation via Linkage Disequilibrium Score Regression and Genomic Restricted Maximum Likelihood. Am J Hum Genet. 2018;102(6):1185-94. <https://doi.org/10.1016/j.ajhg.2018.03.021>.

[10] Mozaffarian D. Dietary and Policy Priorities for Cardiovascular Disease, Diabetes, and Obesity: A Comprehensive Review. Circulation. 2016;133(2):187-225. <https://doi.org/10.1161/circulationaha.115.018585>.

[11] Lourida I, Hannon E, Littlejohns TJ, Langa KM, Hyppönen E, Kuzma E, et al. Association of Lifestyle and Genetic Risk With Incidence of Dementia. Jama. 2019;322(5):430-7. <https://doi.org/10.1001/jama.2019.9879>.

[12] Committee IR. Guidelines for data processing and analysis of the International Physical Activity Questionnaire (IPAQ)-short and long forms. <http://www> ipaq ki se/scoring pdf. 2005.

**Supplementary tables**

| **Table S1. The Hazard ratio (HR) with 95% confidence intervals (CI) of air pollutants for major mental disorder in sensitivity analysis.** | | | | | | | | | | | | | | | |
| --- | --- | --- | --- | --- | --- | --- | --- | --- | --- | --- | --- | --- | --- | --- | --- |
|  | | **Quartile 1** | | **Quartile 2** | | | | **Quartile 3** | | | | **Quartile 4** | | | |
|  |  |  | | HR (95% CI) | | *P* value | | HR (95% CI) | | *P* value | | HR (95% CI) | | *P* value | |
| **PM_2.5_** | Ref. | | 1.06 (0.99-1.13) | | 0.101 | | 1.07 (1.00-1.15) | | 0.040 | | 1.10 (1.04-1.19) | | 0.004 | |  |
| **PM_2.5-10_** | Ref. | | 1.10 (1.03-1.17) | | 0.006 | | 1.02 (0.96-1.10) | | 0.493 | | 1.04 (0.97-1.12) | | 0.250 | |  |
| **NO_2_** | Ref. | | 1.03 (0.96-1.10) | | 0.387 | | 1.09 (1.02-1.17) | | 0.008 | | 1.09 (1.01-1.17) | | 0.023 | |  |
| **NO** | Ref. | | 1.07 (1.00-1.15) | | 0.047 | | 1.09 (1.02-1.17) | | 0.010 | | 1.09 (1.01-1.17) | | 0.020 | |  |
| Notes: The air pollutant concentrations are expressed in quartiles. The model was adjusted for age, gender, education attainment, employment status, smoking frequency per day, drinking frequency per week, 24-hour weighted average noise, distance to major roads, social deprivation, household income, genetic risk of MD, healthy diet score and IPAQ activity. | | | | | | | | | | | | | | |  |

| **Table S2. The Hazard ratio (HR) with 95% confidence intervals (CI) of air pollutants for anxiety (N_cases_=4 929).** | | | | | | | | | | | | | | | |
| --- | --- | --- | --- | --- | --- | --- | --- | --- | --- | --- | --- | --- | --- | --- | --- |
|  | | **Quartile 1** | | **Quartile 2** | | | | **Quartile 3** | | | | **Quartile 4** | | | |
|  |  |  | | HR (95% CI) | | *P* value | | HR (95% CI) | | *P* value | | HR (95% CI) | | *P* value | |
| **PM_2.5_** |  | |  | |  | |  | |  | |  | |  | |  |
| Model 1 | Ref. | | 1.16(1.08-1.26) | | 1.83×10^-4^ | | 1.27(1.17-1.37) | | 5.85×10^-9^ | | 1.34(1.24-1.46) | | 6.82×10^-13^ | |  |
| Model 2 | Ref. | | 1.15(1.06-1.25) | | 4.95×10^-4^ | | 1.24(1.15-1.34) | | 8.77×10^-8^ | | 1.33(1.23-1.45) | | 1.12×10^-11^ | |  |
| Model 3 | Ref. | | 1.10(1.02-1.19) | | 0.019 | | 1.14(1.05-1.23) | | 0.002 | | 1.16(1.06-1.26) | | 0.001 | |  |
| **PM_2.5-10_** | |  | |  | |  | |  | |  | |  | |  | |
| Model 1 | Ref. | | 1.09(1.00-1.17) | | 0.038 | | 1.09(1.01-1.18) | | 0.028 | | 1.08(1.00-1.17) | | 0.052 | |  |
| Model 2 | Ref. | | 1.07(0.99-1.16) | | 0.078 | | 1.08(1.00-1.17) | | 0.047 | | 1.06(0.98-1.16) | | 0.159 | |  |
| Model 3 | Ref. | | 1.04(0.96-1.12) | | 0.333 | | 1.03(0.95-1.12) | | 0.405 | | 1.03(0.94-1.12) | | 0.513 | |  |
| **NO_2_** |  | |  | |  | |  | |  | |  | |  | |  |
| Model 1 | Ref. | | 1.18(1.09-1.27) | | 4.23×10^-5^ | | 1.32(1.22-1.42) | | 5.49×10^-12^ | | 1.33(1.22-1.44) | | 9.89×10^-12^ | |  |
| Model 2 | Ref. | | 1.15(1.06-1.24) | | 4.85×10^-4^ | | 1.29(1.19-1.39) | | 3.50×10^-10^ | | 1.34(1.23-1.46) | | 1.24×10^-11^ | |  |
| Model 3 | Ref. | | 1.1(1.01-1.19) | | 0.021 | | 1.18(1.09-1.28) | | 4.32×10^-5^ | | 1.17(1.07-1.28) | | 0.001 | |  |
| **NO** |  | |  | |  | |  | |  | |  | |  | |  |
| Model 1 | Ref. | | 1.11(1.02-1.20) | | 0.014 | | 1.24(1.15-1.34) | | 8.99×10^-8^ | | 1.24(1.14-1.34) | | 1.36×10^-7^ | |  |
| Model 2 | Ref. | | 1.09(1.01-1.18) | | 0.027 | | 1.22(1.13-1.32) | | 8.43×10^-7^ | | 1.23(1.13-1.34) | | 2.52×10^-6^ | |  |
| Model 3 | Ref. | | 1.04(0.96-1.13) | | 0.312 | | 1.12(1.04-1.22) | | 0.005 | | 1.07(0.98-1.17) | | 0.147 | |  |
| Notes: The air pollutant concentrations are expressed in quartiles. * *P*<0.05.  Model 1: Adjusted for age, gender;  Model 2: Adjusted for age, gender, education attainment, employment status, smoking frequency per day, drinking frequency per week, 24-hour weighted average noise, and distance to major roads;  Model 3: Adjusted for age, gender, education attainment, employment status, smoking frequency per day, drinking frequency per week, 24-hour weighted average noise, distance to major roads, social deprivation, household income, and genetic risk of anxiety. | | | | | | | | | | | | | | |  |
| **Table S3. The Hazard ratio (HR) with 95% confidence intervals (CI) of air pollutants for depression (N_cases_=5 983).** | | | | | | | | | | | | | | | |
|  | | **Quartile 1** | | **Quartile 2** | | | | **Quartile 3** | | | | **Quartile 4** | | | |
|  |  |  | | HR (95% CI) | | *P* value | | HR (95% CI) | | *P* value | | HR (95% CI) | | *P* value | |
| **PM_2.5_** |  | |  | |  | |  | |  | |  | |  | |  |
| Model 1 | Ref. | | 1.17(1.09-1.26) | | 3.21×10^-5^ | | 1.23(1.14-1.32) | | 3.85×10^-8^ | | 1.45(1.35-1.56) | | 5.23×10^-24^ | |  |
| Model 2 | Ref. | | 1.15(1.07-1.24) | | 1.70×10^-4^ | | 1.2(1.11-1.29) | | 1.28×10^-6^ | | 1.45(1.35-1.56) | | 1.23×10^-22^ | |  |
| Model 3 | Ref. | | 1.09(1.01-1.17) | | 0.023 | | 1.08(1.00-1.16) | | 0.049 | | 1.22(1.12-1.32) | | 1.06×10^-6^ | |  |
| **PM_2.5-10_** | |  | |  | |  | |  | |  | |  | |  | |
| Model 1 | Ref. | | 1.15(1.07-1.23) | | 1.11×10^-4^ | | 1.07(1.00-1.15) | | 0.054 | | 1.09(1.02-1.18) | | 0.016 | |  |
| Model 2 | Ref. | | 1.13(1.06-1.22) | | 4.33×10^-4^ | | 1.07(0.99-1.15) | | 0.077 | | 1.09(1.01-1.18) | | 0.029 | |  |
| Model 3 | Ref. | | 1.09(1.02-1.17) | | 0.016 | | 1.01(0.94-1.09) | | 0.799 | | 1.04(0.97-1.13) | | 0.267 | |  |
| **NO_2_** |  | |  | |  | |  | |  | |  | |  | |  |
| Model 1 | Ref. | | 1.15(1.07-1.24) | | 1.06×10^-4^ | | 1.29(1.20-1.38) | | 3.03×10^-12^ | | 1.31(1.22-1.41) | | 5.29×10^-13^ | |  |
| Model 2 | Ref. | | 1.12(1.04-1.20) | | 0.002 | | 1.25(1.16-1.34) | | 9.29×10^-10^ | | 1.33(1.23-1.43) | | 3.76×10^-13^ | |  |
| Model 3 | Ref. | | 1.05(0.98-1.13) | | 0.148 | | 1.12(1.04-1.21) | | 0.002 | | 1.10(1.02-1.20) | | 0.020 | |  |
| **NO** |  | |  | |  | |  | |  | |  | |  | |  |
| Model 1 | Ref. | | 1.17(1.09-1.26) | | 1.39×10^-5^ | | 1.26(1.17-1.35) | | 6.62×10^-10^ | | 1.34(1.25-1.44) | | 2.02×10^-15^ | |  |
| Model 2 | Ref. | | 1.16(1.08-1.25) | | 6.42×10^-5^ | | 1.23(1.15-1.32) | | 1.81×10^-8^ | | 1.35(1.25-1.46) | | 2.00×10^-14^ | |  |
| Model 3 | Ref. | | 1.10(1.02-1.18) | | 0.014 | | 1.12(1.04-1.20) | | 0.004 | | 1.14(1.05-1.24) | | 0.001 | |  |
| Notes: The air pollutant concentrations are expressed in quartiles. * *P*<0.05.  Model 1: Adjusted for age, gender;  Model 2: Adjusted for age, gender, education attainment, employment status, smoking frequency per day, drinking frequency per week, 24-hour weighted average noise, and distance to major roads;  Model 3: Adjusted for age, gender, education attainment, employment status, smoking frequency per day, drinking frequency per week, 24-hour weighted average noise, distance to major roads, social deprivation, household income, and genetic risk of depression. | | | | | | | | | | | | | | |  |

| **Table S4. The Hazard ratio (HR) with 95% confidence intervals (CI) of air pollutants for schizophrenia (N_cases_=243).** | | | | | | | | | | | | | | | |
| --- | --- | --- | --- | --- | --- | --- | --- | --- | --- | --- | --- | --- | --- | --- | --- |
|  | | **Quartile 1** | | **Quartile 2** | | | | **Quartile 3** | | | | **Quartile 4** | | | |
|  |  |  | | HR (95% CI) | | *P* value | | HR (95% CI) | | *P* value | | HR (95% CI) | | *P* value | |
| **PM_2.5_** |  | |  | |  | |  | |  | |  | |  | |  |
| Model 1 | Ref. | | 1.38(0.95-2.02) | | 0.091 | | 1.34(0.91-1.97) | | 0.136 | | 2.03(1.41-2.91) | | 1.27×10^-4^ | |  |
| Model 2 | Ref. | | 1.4(0.96-2.04) | | 0.081 | | 1.33(0.90-1.95) | | 0.152 | | 1.94(1.33-2.82) | | 0.001 | |  |
| Model 3 | Ref. | | 1.27(0.87-1.86) | | 0.211 | | 1.11(0.75-1.64) | | 0.609 | | 1.44(0.97-2.14) | | 0.070 | |  |
| **PM_2.5-10_** | |  | |  | |  | |  | |  | |  | |  | |
| Model 1 | Ref. | | 1.38(0.97-1.96) | | 0.074 | | 1.03(0.70-1.51) | | 0.894 | | 1.43(1.00-2.05) | | 0.047 | |  |
| Model 2 | Ref. | | 1.34(0.94-1.91) | | 0.100 | | 0.97(0.66-1.44) | | 0.894 | | 1.23(0.83-1.81) | | 0.300 | |  |
| Model 3 | Ref. | | 1.26(0.89-1.79) | | 0.200 | | 0.89(0.60-1.31) | | 0.549 | | 1.15(0.78-1.70) | | 0.484 | |  |
| **NO_2_** |  | |  | |  | |  | |  | |  | |  | |  |
| Model 1 | Ref. | | 1.12(0.76-1.64) | | 0.578 | | 1.47(1.01-2.12) | | 0.041 | | 2.10(1.47-3.00) | | 4.12×10^-5^ | |  |
| Model 2 | Ref. | | 1.10(0.75-1.62) | | 0.619 | | 1.44(0.99-2.09) | | 0.055 | | 2.06(1.42-2.99) | | 1.29×10^-4^ | |  |
| Model 3 | Ref. | | 1.00(0.68-1.48) | | 0.995 | | 1.22(0.83-1.78) | | 0.311 | | 1.57(1.06-2.34) | | 0.026 | |  |
| **NO** |  | |  | |  | |  | |  | |  | |  | |  |
| Model 1 | Ref. | | 1.22(0.84-1.78) | | 0.297 | | 1.28(0.88-1.86) | | 0.205 | | 1.85(1.30-2.64) | | 0.001 | |  |
| Model 2 | Ref. | | 1.22(0.83-1.77) | | 0.310 | | 1.25(0.86-1.83) | | 0.243 | | 1.72(1.17-2.52) | | 0.006 | |  |
| Model 3 | Ref. | | 1.11(0.76-1.62) | | 0.591 | | 1.07(0.73-1.56) | | 0.747 | | 1.29(0.87-1.92) | | 0.204 | |  |
| Notes: The air pollutant concentrations are expressed in quartiles. * *P*<0.05.  Model 1: Adjusted for age, gender;  Model 2: Adjusted for age, gender, education attainment, employment status, smoking frequency per day, drinking frequency per week, 24-hour weighted average noise, and distance to major roads;  Model 3: Adjusted for age, gender, education attainment, employment status, smoking frequency per day, drinking frequency per week, 24-hour weighted average noise, distance to major roads, social deprivation, household income, and genetic risk of schizophrenia. | | | | | | | | | | | | | | |  |

| **Table S5. The Hazard ratio (HR) with 95% confidence intervals (CI) of air pollutants for bipolar disorder (N_cases_=246).** | | | | | | | | | | | | | | | |
| --- | --- | --- | --- | --- | --- | --- | --- | --- | --- | --- | --- | --- | --- | --- | --- |
|  | | **Quartile 1** | | **Quartile 2** | | | | **Quartile 3** | | | | **Quartile 4** | | | |
|  |  |  | | HR (95% CI) | | *P* value | | HR (95% CI) | | *P* value | | HR (95% CI) | | *P* value | |
| **PM_2.5_** |  | |  | |  | |  | |  | |  | |  | |  |
| Model 1 | Ref. | | 1.25(0.87-1.81) | | 0.233 | | 1.47(1.02-2.11) | | 0.037 | | 1.60(1.11-2.30) | | 0.012 | |  |
| Model 2 | Ref. | | 1.28(0.88-1.85) | | 0.193 | | 1.50(1.04-2.15) | | 0.029 | | 1.58(1.08-2.30) | | 0.018 | |  |
| Model 3 | Ref. | | 1.18(0.81-1.71) | | 0.386 | | 1.26(0.87-1.83) | | 0.217 | | 1.17(0.78-1.74) | | 0.445 | |  |
| **PM_2.5-10_** | |  | |  | |  | |  | |  | |  | |  | |
| Model 1 | Ref. | | 0.82(0.58-1.16) | | 0.269 | | 0.82(0.57-1.16) | | 0.264 | | 0.97(0.69-1.36) | | 0.856 | |  |
| Model 2 | Ref. | | 0.82(0.58-1.15) | | 0.245 | | 0.80(0.56-1.14) | | 0.222 | | 0.88(0.61-1.28) | | 0.509 | |  |
| Model 3 | Ref. | | 0.78(0.55-1.10) | | 0.158 | | 0.73(0.51-1.05) | | 0.088 | | 0.83(0.57-1.20) | | 0.314 | |  |
| **NO_2_** |  | |  | |  | |  | |  | |  | |  | |  |
| Model 1 | Ref. | | 1.30(0.9-1.87) | | 0.161 | | 1.34(0.92-1.93) | | 0.124 | | 1.81(1.27-2.59) | | 0.001 | |  |
| Model 2 | Ref. | | 1.31(0.91-1.89) | | 0.145 | | 1.36(0.94-1.97) | | 0.105 | | 1.84(1.27-2.67) | | 0.001 | |  |
| Model 3 | Ref. | | 1.21(0.84-1.75) | | 0.301 | | 1.15(0.79-1.68) | | 0.473 | | 1.36(0.91-2.02) | | 0.132 | |  |
| **NO** |  | |  | |  | |  | |  | |  | |  | |  |
| Model 1 | Ref. | | 1.13(0.79-1.61) | | 0.514 | | 1.04(0.72-1.51) | | 0.834 | | 1.57(1.11-2.21) | | 0.011 | |  |
| Model 2 | Ref. | | 1.14(0.80-1.64) | | 0.465 | | 1.06(0.73-1.54) | | 0.767 | | 1.59(1.10-2.31) | | 0.014 | |  |
| Model 3 | Ref. | | 1.07(0.74-1.53) | | 0.718 | | 0.92(0.63-1.34) | | 0.661 | | 1.24(0.85-1.83) | | 0.270 | |  |
| *Notes: The air pollutant concentrations are expressed in quartiles. * *P*<0.05.  Model 1: Adjusted for age, gender;  Model 2: Adjusted for age, gender, education attainment, employment status, smoking frequency per day, drinking frequency per week, 24-hour weighted average noise, and distance to major roads;  Model 3: Adjusted for age, gender, education attainment, employment status, smoking frequency per day, drinking frequency per week, 24-hour weighted average noise, distance to major roads, social deprivation, household income, and genetic risk of bipolar disorder. | | | | | | | | | | | | | | |  |

| **Table S6.** Hazard ratio with 95% confidence intervals for MD stratified by household income. | | | |
| --- | --- | --- | --- |
|  | **Low income** | **Moderate income** | **High income** |
|  | **N events= 5,347** | **N events= 2,155** | **N events= 1,502** |
|  | **HR (95% CI)** | **HR (95% CI)** | **HR (95% CI)** |
| **NO_2_** |  |  |  |
| Q1 | 1(Ref.) | 1(Ref.) | 1(Ref.) |
| Q2 | 1.03(0.95-1.12) | 1.05(0.93-1.19) | 1.07(0.93-1.24) |
| Q3 | **1.11(1.03-1.21)** | 1.09(0.97-1.23) | 1.11(0.97-1.29) |
| Q4 | **1.14(1.05-1.25)** | 1.12(0.98-1.28) | 1.06(0.91-1.24) |
| **P for trend** | 4.66×10^-4^ | 0.078 | 0.302 |
| **P for Interaction:** 0.069 | | | |
| **NO** |  |  |  |
| Q1 | 1(Ref.) | 1(Ref.) | 1(Ref.) |
| Q2 | 1.10(1.01-1.20) | 1.08(0.95-1.22) | 1.03(0.90-1.19) |
| Q3 | **1.13(1.04-1.23)** | **1.14(1.01-1.28)** | 1.01(0.87-1.17) |
| Q4 | **1.09(1.00-1.19)** | 1.13(0.99-1.29) | 1.13(0.97-1.33) |
| **P for trend** | 0.042 | 0.043 | 0.203 |
| **P for Interaction:** 0.874 | | | |
| **PM_2.5_** |  |  |  |
| Q1 | 1(Ref.) | 1(Ref.) | 1(Ref.) |
| Q2 | 1.08(1.00-1.18) | 1.11(0.98-1.25) | 1.06(0.92-1.22) |
| Q3 | **1.08(1.00-1.18)** | **1.17(1.04-1.33)** | 1.03(0.89-1.19) |
| Q4 | **1.15(1.06-1.26)** | **1.15(1.01-1.31)** | **1.21(1.04-1.41)** |
| **P for trend** | 0.002 | 0.021 | 0.034 |
| **P for Interaction:** 0.610 | | | |
| **PM_2.5-10_** |  |  |  |
| Q1 | 1(Ref.) | 1(Ref.) | 1(Ref.) |
| Q2 | 1.06(0.98-1.15) | **1.22(1.08-1.37)** | 1.11(0.96-1.29) |
| Q3 | 1.03(0.95-1.11) | 1.06(0.94-1.20) | 1.05(0.91-1.22) |
| Q4 | 1.04(0.95-1.12) | **1.14(1.00-1.30)** | 1.08(0.93-1.26) |
| **P for trend** | 0.602 | 0.210 | 0.450 |
| **P for Interaction:** 0.517 | | | |
| * The air pollutant concentrations are expressed in quartiles. The model was adjusted for age, gender, education attainment, employment status, smoking frequency per day, drinking frequency per week, 24-hour weighted average noise, distance to major roads, social deprivation and genetic risk of MD. The statistically significant associations are bolded. | | | |

| **Table S7.** Hazard ratio with 95% confidence intervals for MD stratified by social deprivation. | | | |
| --- | --- | --- | --- |
|  | **Low deprivation** | **Moderate deprivation** | **High deprivation** |
|  | **N events= 2,586** | **N events= 2,840** | **N events= 3,578** |
|  | **HR (95% CI)** | **HR (95% CI)** | **HR (95% CI)** |
| **NO_2_** |  |  |  |
| Q1 | 1(Ref.) | 1(Ref.) | 1(Ref.) |
| Q2 | 1.04(0.95-1.15) | 1.03(0.93-1.14) | 1.05(0.92-1.19) |
| Q3 | **1.12(1.01-1.24)** | 1.08(0.97-1.19) | 1.11(0.99-1.26) |
| Q4 | 1.13(0.97-1.31) | **1.16(1.04-1.30)** | 1.11(0.99-1.24) |
| **P for trend** | 0.020 | 0.006 | 0.065 |
| **P for Interaction:** 0.664 | | | |
| **NO** |  |  |  |
| Q1 | 1(Ref.) | 1(Ref.) | 1(Ref.) |
| Q2 | 1.04(0.94-1.15) | **1.17(1.05-1.30)** | 1.04(0.92-1.17) |
| Q3 | 1.03(0.93-1.14) | **1.18(1.07-1.31)** | 1.11(0.99-1.24) |
| Q4 | 1.06(0.93-1.21) | **1.14(1.02-1.29)** | 1.10(0.98-1.23) |
| **P for trend** | 0.392 | 0.011 | 0.062 |
| **P for Interaction:** 0.622 | | | |
| **PM_2.5_** |  |  |  |
| Q1 | 1(Ref.) | 1(Ref.) | 1(Ref.) |
| Q2 | **1.12(1.01-1.23)** | 1.06(0.96-1.18) | 1.06(0.94-1.21) |
| Q3 | 1.09(0.98-1.21) | 1.10(0.99-1.21) | 1.08(0.96-1.22) |
| Q4 | **1.16(1.02-1.31)** | **1.18(1.06-1.32)** | **1.14(1.02-1.28)** |
| **P for trend** | 0.024 | 0.002 | 0.013 |
| **P for Interaction:** 0.838 | | | |
| **PM_2.5-10_** |  |  |  |
| Q1 | 1(Ref.) | 1(Ref.) | 1(Ref.) |
| Q2 | **1.16(1.05-1.29)** | 1.00(0.91-1.11) | **1.16(1.05-1.28)** |
| Q3 | 1.06(0.94-1.18) | 0.97(0.87-1.08) | 1.08(0.98-1.19) |
| Q4 | 1.11(0.99-1.25) | 1.05(0.94-1.17) | 1.05(0.95-1.17) |
| **P for trend** | 0.155 | 0.548 | 0.797 |
| **P for Interaction:** 0.205 | | | |
| * The air pollutant concentrations are expressed in quartiles. The model was adjusted for age, gender, education attainment, employment status, smoking frequency per day, drinking frequency per week, 24-hour weighted average noise, distance to major roads, household income and genetic risk of MD. The statistically significant associations are bolded. | | | |

| **Table S8.** Hazard ratio with 95% confidence intervals for MD stratified by genetic risk. | | | |
| --- | --- | --- | --- |
|  | **Low genetic risk** | **Moderate genetic risk** | **High genetic risk** |
|  | **N events= 2-520** | **N events= 2-965** | **N events= 3-519** |
|  | **HR (95% CI)** | **HR (95% CI)** | **HR (95% CI)** |
| **NO_2_** |  |  |  |
| Q1 | 1(Ref.) | 1(Ref.) | 1(Ref.) |
| Q2 | 1.09(0.97-1.22) | 0.97(0.87-1.08) | 1.07(0.97-1.18) |
| Q3 | 1.07(0.96-1.20) | **1.12(1.01-1.24)** | **1.13(1.02-1.24)** |
| Q4 | **1.17(1.04-1.32)** | 1.03(0.92-1.16) | **1.17(1.05-1.29)** |
| **P for trend** | 0.024 | 0.161 | 0.002 |
| **P for Interaction:** 0.558 | | | |
| **NO** |  |  |  |
| Q1 | 1(Ref.) | 1(Ref.) | 1(Ref.) |
| Q2 | 1.11(0.99-1.24) | 1.00(0.90-1.11) | **1.13(1.03-1.25)** |
| Q3 | 1.11(0.99-1.24) | 1.08(0.98-1.2) | **1.14(1.03-1.26)** |
| Q4 | 1.09(0.97-1.24) | 1.05(0.93-1.17) | **1.16(1.05-1.29)** |
| **P for trend** | 0.176 | 0.219 | 0.008 |
| **P for Interaction:** 0.404 | | | |
| **PM_2.5_** |  |  |  |
| Q1 | 1(Ref.) | 1(Ref.) | 1(Ref.) |
| Q2 | 1.09(0.97-1.22) | 1.07(0.96-1.19) | 1.10(0.99-1.21) |
| Q3 | **1.13(1.01-1.27)** | 1.04(0.94-1.16) | **1.12(1.01-1.23)** |
| Q4 | **1.24(1.10-1.40)** | 1.09(0.98-1.22) | **1.17(1.05-1.29)** |
| **P for trend** | 4.25×10^-4^ | 0.186 | 0.005 |
| **P for Interaction:** 0.608 | | | |
| **PM_2.5-10_** | |  |  |
| Q1 | 1(Ref.) | 1(Ref.) | 1(Ref.) |
| Q2 | 1.09(0.97-1.22) | 1.07(0.97-1.18) | **1.16(1.06-1.27)** |
| Q3 | **1.12(1.01-1.26)** | 1.00(0.9-1.11) | 1.02(0.93-1.12) |
| Q4 | 1.06(0.94-1.20) | 1.07(0.96-1.2) | 1.06(0.96-1.18) |
| **P for trend** | 0.233 | 0.415 | 0.738 |
| **P for Interaction:** 0.709 | | | |
| * The air pollutant concentrations are expressed in quartiles. The model was adjusted for age, gender, education attainment, employment status, smoking frequency per day, drinking frequency per week, 24-hour weighted average noise, distance to major roads, household income and social deprivation. The statistically significant associations are bolded. | | | |

| **Table S9. Joint effect of social deprivation and air pollution on the risk of major mental disorder.** | | | | | | | | | |
| --- | --- | --- | --- | --- | --- | --- | --- | --- | --- |
| **Social deprivation** |  | **PM_2.5_** | | **PM_2.5-10_** |  | **NO_2_** |  | **NO** |  |
|  |  | **HR (95% CI)** | ***P* value** | **HR (95% CI)** | ***P* value** | **HR (95% CI)** | ***P* value** | **HR (95% CI)** | ***P* value** |
| Low | Quartile1 | Ref. |  | Ref. |  | Ref. |  | Ref. |  |
| Low | Quartile2 | 1.11(1.01-1.22) | 0.039 | 1.16(1.05-1.28) | 0.004 | 1.03(0.94-1.13) | 0.54 | 1.03(0.93-1.14) | 0.566 |
| Low | Quartile3 | 1.08(0.97-1.20) | 0.157 | 1.06(0.95-1.19) | 0.304 | 1.11(1.01-1.23) | 0.037 | 1.02(0.92-1.13) | 0.691 |
| Low | Quartile4 | 1.16(1.02-1.32) | 0.019 | 1.13(1.01-1.26) | 0.033 | 1.15(1.00-1.33) | 0.057 | 1.08(0.95-1.22) | 0.242 |
| Moderate | Quartile1 | 1.05(0.95-1.16) | 0.36 | 1.13(1.02-1.25) | 0.015 | 1.05(0.95-1.15) | 0.376 | 0.96(0.87-1.07) | 0.468 |
| Moderate | Quartile2 | 1.11(1.01-1.23) | 0.035 | 1.14(1.03-1.26) | 0.011 | 1.08(0.98-1.19) | 0.141 | 1.13(1.02-1.24) | 0.019 |
| Moderate | Quartile3 | 1.15(1.04-1.27) | 0.005 | 1.10(0.99-1.23) | 0.064 | 1.13(1.02-1.24) | 0.014 | 1.14(1.04-1.26) | 0.008 |
| Moderate | Quartile4 | 1.26(1.13-1.39) | 1.84×10^-5^ | 1.21(1.09-1.34) | 4.50×10^-4^ | 1.23(1.11-1.37) | 1.05×10^-4^ | 1.12(1.01-1.25) | 0.033 |
| High | Quartile1 | 1.27(1.12-1.43) | 1.16×10^-4^ | 1.31(1.18-1.46) | 5.48×10^-7^ | 1.25(1.11-1.41) | 3.06×10^-4^ | 1.24(1.10-1.39) | 2.96×10^-4^ |
| High | Quartile2 | 1.36(1.22-1.51) | 7.72×10^-9^ | 1.52(1.38-1.67) | 6.03×10^-17^ | 1.33(1.20-1.47) | 7.27×10^-8^ | 1.29(1.17-1.43) | 8.29×10^-7^ |
| High | Quartile3 | 1.38(1.26-1.51) | 1.67×10^-11^ | 1.40(1.28-1.54) | 2.83×10^-12^ | 1.41(1.28-1.54) | 6.20×10^-13^ | 1.38(1.25-1.51) | 2.20×10^-11^ |
| High | Quartile4 | 1.43(1.31-1.56) | 3.36×10^-16^ | 1.35(1.22-1.48) | 3.26×10^-9^ | 1.36(1.25-1.48) | 4.30×10^-13^ | 1.33(1.22-1.46) | 2.89×10^-10^ |

| **Table S10. Joint effect of household and air pollution on the risk of major mental disorder.** | | | | | | | | | |  |
| --- | --- | --- | --- | --- | --- | --- | --- | --- | --- | --- |
| **Household income** |  | **PM_2.5_** | | **PM_2.5-10_** |  | **NO_2_** |  | **NO** |  | |
|  |  | **HR (95% CI)** | ***P* value** | **HR (95% CI)** | ***P* value** | **HR (95% CI)** | ***P* value** | **HR (95% CI)** | ***P* value** | |
| High | Quartile1 | Ref. |  | Ref. |  | Ref. |  | Ref. |  | |
| High | Quartile2 | 1.05(0.91-1.20) | 0.533 | 1.11(0.96-1.28) | 0.160 | 1.06(0.92-1.22) | 0.398 | 1.02(0.89-1.17) | 0.789 | |
| High | Quartile3 | 0.99(0.86-1.15) | 0.927 | 1.03(0.90-1.20) | 0.643 | 1.10(0.95-1.27) | 0.201 | 0.98(0.85-1.13) | 0.824 | |
| High | Quartile4 | 1.15(1.00-1.33) | 0.049 | 1.09(0.95-1.26) | 0.231 | 1.02(0.89-1.18) | 0.762 | 1.10(0.95-1.27) | 0.190 | |
| Moderate | Quartile1 | 1.26(1.11-1.44) | 4.77×10^-4^ | 1.27(1.11-1.45) | 3.99×10^-4^ | 1.31(1.15-1.49) | 4.16×10^-5^ | 1.26(1.11-1.43) | 4.38×10^-4^ | |
| Moderate | Quartile2 | 1.38(1.21-1.57) | 7.57×10^-7^ | 1.54(1.36-1.76) | 4.23×10^-11^ | 1.36(1.20-1.55) | 2.43×10^-6^ | 1.34(1.18-1.53) | 5.13×10^-6^ | |
| Moderate | Quartile3 | 1.45(1.28-1.65) | 7.57×10^-9^ | 1.34(1.17-1.53) | 2.29×10^-5^ | 1.41(1.24-1.60) | 2.03×10^-7^ | 1.41(1.25-1.60) | 7.45×10^-8^ | |
| Moderate | Quartile4 | 1.42(1.25-1.61) | 1.13×10^-7^ | 1.42(1.25-1.63) | 2.04×10^-7^ | 1.42(1.25-1.62) | 1.02×10^-7^ | 1.38(1.22-1.57) | 7.44×10^-7^ | |
| Low | Quartile1 | 1.70(1.51-1.91) | 7.13×10^-19^ | 1.82(1.62-2.05) | 3.84×10^-23^ | 1.71(1.52-1.92) | 2.27×10^-19^ | 1.66(1.48-1.87) | 6.29×10^-18^ | |
| Low | Quartile2 | 1.85(1.65-2.08) | 2.94×10^-26^ | 1.94(1.73-2.18) | 1.51×10^-28^ | 1.77(1.58-1.99) | 7.86×10^-23^ | 1.85(1.65-2.06) | 9.92×10^-27^ | |
| Low | Quartile3 | 1.86(1.67-2.09) | 2.93×10^-27^ | 1.88(1.67-2.12) | 1.28×10^-25^ | 1.93(1.72-2.16) | 3.88×10^-30^ | 1.91(1.71-2.13) | 5.60×10^-30^ | |
| Low | Quartile4 | 1.99(1.78-2.23) | 6.72×10^-33^ | 1.88(1.67-2.12) | 5.79×10^-25^ | 1.99(1.77-2.23) | 1.50×10^-31^ | 1.84(1.65-2.06) | 2.42×10^-26^ | |

| **Table S11. Joint effect of genetic risk and air pollution on the risk of major mental disorder.** | | | | | | | | | |  |
| --- | --- | --- | --- | --- | --- | --- | --- | --- | --- | --- |
| **Genetic Risk** |  | **PM_2.5_** | | **PM_2.5-10_** |  | **NO_2_** |  | **NO** |  | |
|  |  | **HR (95% CI)** | ***P* value** | **HR (95% CI)** | ***P* value** | **HR (95% CI)** | ***P* value** | **HR (95% CI)** | ***P* value** | |
| Low | Quartile1 | Ref. |  | Ref. |  | Ref. |  | Ref. |  | |
| Low | Quartile2 | 1.10(0.98-1.23) | 0.113 | 1.09(0.98-1.22) | 0.127 | 1.10(0.98-1.23) | 0.094 | 1.12(1.00-1.25) | 0.058 | |
| Low | Quartile3 | 1.14(1.02-1.28) | 0.024 | 1.12(1.00-1.26) | 0.041 | 1.09(0.97-1.22) | 0.161 | 1.12(1.00-1.25) | 0.052 | |
| Low | Quartile4 | 1.24(1.11-1.39) | 2.10×10^-4^ | 1.06(0.94-1.19) | 0.338 | 1.17(1.04-1.31) | 0.007 | 1.10(0.98-1.24) | 0.097 | |
| Moderate | Quartile1 | 1.25(1.12-1.40) | 8.98×10^-5^ | 1.21(1.08-1.35) | 6.20×10^-4^ | 1.24(1.11-1.39) | 1.63×10^-4^ | 1.23(1.10-1.38) | 2.69×10^-4^ | |
| Moderate | Quartile2 | 1.32(1.18-1.48) | 8.23×10^-7^ | 1.28(1.15-1.43) | 4.90×10^-6^ | 1.19(1.07-1.33) | 0.002 | 1.22(1.10-1.37) | 3.78×10^-4^ | |
| Moderate | Quartile3 | 1.29(1.15-1.44) | 7.50×10^-6^ | 1.19(1.06-1.33) | 0.002 | 1.37(1.23-1.53) | 8.55×10^-9^ | 1.32(1.18-1.47) | 6.83×10^-7^ | |
| Moderate | Quartile4 | 1.34(1.20-1.49) | 3.39×10^-7^ | 1.27(1.14-1.42) | 1.80×10^-5^ | 1.25(1.12-1.4) | 1.07×10^-4^ | 1.26(1.13-1.41) | 5.400×10^-5^ | |
| High | Quartile1 | 1.38(1.23-1.54) | 1.96×10^-8^ | 1.36(1.22-1.51) | 1.69×10^-8^ | 1.35(1.21-1.51) | 1.23×10^-7^ | 1.32(1.18-1.48) | 8.39×10^-7^ | |
| High | Quartile2 | 1.51(1.35-1.68) | 9.43×10^-14^ | 1.58(1.42-1.75) | 5.33×10^-18^ | 1.43(1.29-1.60) | 4.99×10^-11^ | 1.50(1.34-1.66) | 1.80×10^-13^ | |
| High | Quartile3 | 1.54(1.38-1.71) | 2.85×10^-15^ | 1.39(1.25-1.55) | 7.63×10^-10^ | 1.51(1.36-1.68) | 1.55×10^-14^ | 1.50(1.35-1.67) | 5.15×10^-14^ | |
| High | Quartile4 | 1.63(1.46-1.81) | 3.21×10^-19^ | 1.46(1.31-1.63) | 2.44×10^-12^ | 1.59(1.43-1.77) | 1.41×10^-17^ | 1.54(1.39-1.72) | 2.29×10^-15^ | |

| **Table S12.** The genetic correlations between mental disorders. | | | | |
| --- | --- | --- | --- | --- |
|  | Depression | Anxiety disorder | Bipolar disorder | Schizophrenia |
| Depression | 1 | 0.9863 | 0.5033 | 0.3983 |
| Anxiety disorder | 0.9863 | 1 | 0.2659 | 0.3288 |
| Bipolar disorder | 0.5033 | 0.2659 | 1 | 0.6986 |
| Schizophrenia | 0.3983 | 0.3288 | 0.6986 | 1 |

| **Table S13.** The weight for PRS calculation based on principal component analysis. | | | | |
| --- | --- | --- | --- | --- |
|  | Depression | Anxiety disorder | Bipolar disorder | Schizophrenia |
| Weights | 0.2375711 | 0.2636269 | 0.2493509 | 0.2495132 |
| The first 2 PCs with eigenvalues ​​>1 were used to calculate the PRS for major mental disorder, explaining a total of 92.5% of the variance. | | | | |
